# Leveraging Numerical Simulation Technology to Advance Drug Preparation: A Comprehensive Review of Application Scenarios and Cases

**DOI:** 10.3390/pharmaceutics16101304

**Published:** 2024-10-07

**Authors:** Qifei Gu, Huichao Wu, Xue Sui, Xiaodan Zhang, Yongchao Liu, Wei Feng, Rui Zhou, Shouying Du

**Affiliations:** 1 College of Chinese Materia Medica, Beijing University of Chinese Medicine, Beijing 102488, China; gqf21585@163.com (Q.G.); 20230935242@bucm.edu.cn (X.S.); 20220935231@bucm.edu.cn (X.Z.); 18706386896@163.com (Y.L.); 2 School of Traditional Chinese Medicine, Beijing University of Chinese Medicine, Beijing 102488, China; wuhuichao@bucm.edu.cn; 3 Institute of Ethnic Medicine and Pharmacy, Beijing University of Chinese Medicine, Beijing 102488, China; 4 Wangjing Hospital, China Academy of Traditional Chinese Medicine, Beijing 100102, China; 13611147987@163.com

**Keywords:** numerical simulation, mechanistic modeling, pharmaceutics, quality by design, interdisciplinarity

## Abstract

Background/Objectives: Numerical simulation plays an important role in pharmaceutical preparation recently. Mechanistic models, as a type of numerical model, are widely used in the study of pharmaceutical preparations. Mechanistic models are based on a priori knowledge, i.e., laws of physics, chemistry, and biology. However, due to interdisciplinary reasons, pharmacy researchers have greater difficulties in using computer models. Methods: In this paper, we highlight the application scenarios and examples of mechanistic modelling in pharmacy research and provide a reference for drug researchers to get started. Results: By establishing a suitable model and inputting preparation parameters, researchers can analyze the drug preparation process. Therefore, mechanistic models are effective tools to optimize the preparation parameters and predict potential quality problems of the product. With product quality parameters as the ultimate goal, the experiment design is optimized by mechanistic models. This process emphasizes the concept of quality by design. Conclusions: The use of numerical simulation saves experimental cost and time, and speeds up the experimental process. In pharmacy experiments, part of the physical information and the change processes are difficult to obtain, such as the mechanical phenomena during tablet compression and the airflow details in the nasal cavity. Therefore, it is necessary to predict the information and guide the formulation with the help of mechanistic models.

## 1. Introduction

The quality by design (QbD) philosophy recognized that quality should be designed into a product, and that most quality crises and problems relate to the way in which a product was designed in the first place [1]. The objectives of implementing QbD for pharmaceutics are summarized in four points [2]:

(1) To achieve meaningful product quality specifications that are based on clinical performance.
(2) To increase process capability and reduce product variability and defects by enhancing product and process design, understanding, and controlling.
(3) To increase product development and manufacturing efficiencies.
(4) To enhance root cause analysis and post-approval change management.

**Figure 1:**
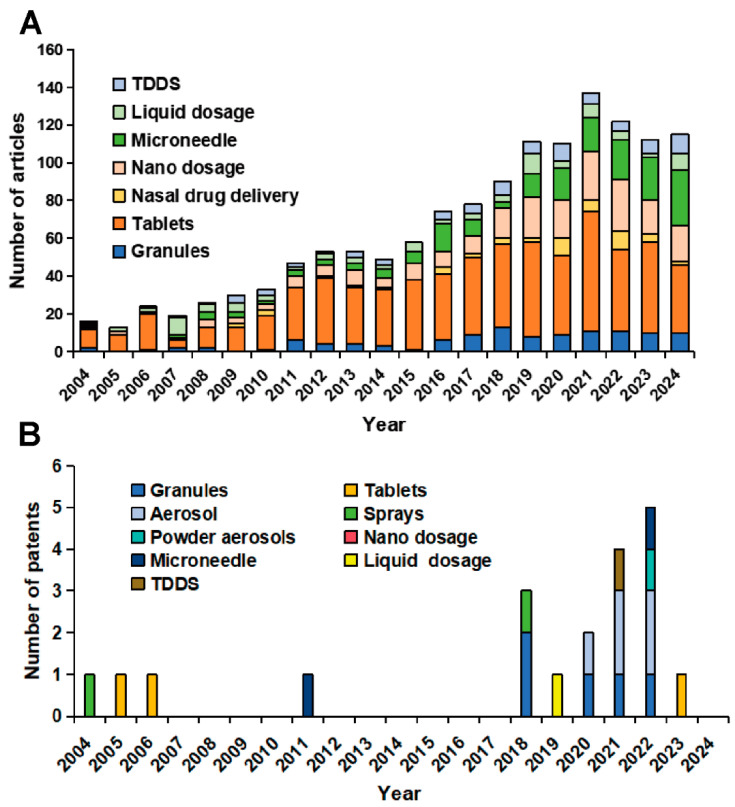
Number of (**A**) articles and (**B**) patents on numerical simulation applied to pharmaceutics in the last 20 years.

The use of numerical simulation in pharmaceutical preparation studies can achieve the above QbD objectives (2) and (3). Advantages of numerical simulation include low cost of trial and error, flexibility of parameter tuning, and wide access of information. Through modeling, we can evaluate the process design in multiple dimensions, including key process parameters [3,4,5] and critical material properties [6,7,8]. We can select experimental parameters, judge the feasibility of the parameters, and then adjust the experimental design by simulation. We can also explore potential failure mechanisms and analyze macro- and micro-physical mechanisms to reveal the underlying causes of the design, so as to improve the efficiency of pharmaceutical product development [9,10]. For example, the simulation of the tablet pressing can predict the potential risk of cracking and explore the causes of cracking. With simulation results, the excipient components and tablet pressing design can be adjusted. In addition, while searching for the optimal process based on the QbD concept, the experimental workload is huge, which leads to a significant increase in the consumption of time and labor. Numerical simulation is a good strategy to solve this problem. The process of evaluating the optimal drug preparation conditions by numerical simulation is fast compared to experiments and reduces the consumption of experimental reagents. Meanwhile, for dosage forms such as nasal drug delivery and microneedle drug delivery, researchers can establish human physiological structure models for simulation, which intuitively express the drug delivery into the human body. The application of numerical simulation increases product development and manufacturing efficiency. Furthermore, the application of numerical simulation has its own irreplaceable advantages in the production process of pharmaceutical preparations. Firstly, in the use of pharmaceutical machinery, we cannot directly observe the state of drug dosage form manufacturing inside the machine such as stress and deformation during the manufacturing process. Secondly, the microscopic changes in the pharmaceutical preparation process are also impossible to be observed with the new dosage forms tend to be at the micrometer and nanometer level. The above two problems can be solved by numerical simulation in the analysis of forces, motion laws, and interactions among the three phases of solid, liquid, and gas. The numerical simulation provides new perspectives and tools for pharmaceutical preparation research. In the last 20 years, there has been an increasing number of studies applying numerical modelling in pharmaceutics in Figure 1A. The largest proportion of application is for tablets and the smallest is for nasal administration. The number of patents is shown in Figure 1B in the last 20 years in the field of simulation-related pharmaceutics, which were searched on the WIPO website and the Chinese National Intellectual Property Administration website. Overall, the use of numerical simulation in pharmaceutics has shown a growing trend over the past 20 years. There is still a lot of potential for future research.

This paper reviews the widely used mechanistic models in pharmaceutical preparation research. Mechanistic models are accurate mathematical models constructed on the basis of the internal mechanisms of an object or production process and the principles of transfer of material flows. Mechanistic models rely on a variety of equations and laws such as the mass conservation equation and the energy conservation equation to enable computational simulation of any object or process. In the field of pharmaceutical simulation, commonly used mechanistic models are based on the theories of the finite element method (FEM), the finite volume method (FVM), finite-difference methods (FDM), and the discrete element model (DEM). The population balance model (PBM) and the computational fluid dynamics (CFD) are two methods for mechanistic models [11,12,13]. However, non-mechanistic models are mainly represented by artificial intelligence (AI) and neural networks (NN), which rely on large-scale data collected from sources such as the Internet and construct decision trees through data processing and learning [9,14,15].

The FEM, FDM and FVM are fundamental tools for solving partial differential equations. Among them, the FEM is particularly suitable for the mechanical analysis of elastic and elastoplastic solids [16] such as the process of tablet pressing. The FEM can show stress concentrations at the fracture point, but cannot demonstrate the fracture process when simulating an object under stress. FDM is the main solution method for fluid mechanics problems. FVM divides the object into a finite number of volume cells to perform a regional discretization and solve each volume cell. Further, the DEM is used for the solution of discrete systems and is mainly used to calculate the particle–-particle and particle-wall contact interactions. The DEM is capable of modeling fracture phenomena and excels in the analysis of low relative density and low stress states of loose powders such as powder flow [17,18]. In pharmaceutical preparation studies, the DEM is mainly used for the simulation of granulation, formulation and nasal spray processes. When using the DEM, it is important to select contact model and input accurate model parameters, as described in detail by Yeom et al. [19]. Nevertheless, the DEM has some limitations such as the particle computation volume and the long simulation time. CFD has its own advantages to analyze fluid issues. CFD is capable of handling the flow of gases and liquids as well as the continuous flow of granular powders. Based on statistical principles, the PBM is used to simulate the processes of nucleation, growth, dissolution, dispersion, aggregation and fragmentation of particles or bubbles. In pharmaceutical research, the PBM can simulate crystallization, precipitation, spraying, granulation and flow of aerosols [7,20,21].

Since the 1960s, computer programs have been developed for numerical simulation. Among the many commercial simulation software, a few commercial software is commonly used in the scientific research field. In 1963, the STASYS program was written, which was the predecessor of ANSYS. In 1979, it was run on minicomputers. ANSYS is a full-function large-scale general-purpose FEM software to analyze the integration of multiphysics fields such as structural, fluid, electric, magnetic, and acoustic fields. ANSYS covers most of the algorithms to solve many problems in mechanics, fluids, particle motion, electric and magnetic fields, etc. ANSYS is more widely used in mechanics and is commonly used for the evaluation of stress-strain during tablet compression, the prediction of hydrodynamics for airway drug delivery and the simulation of microneedle hydrodynamics. In 1978, the original versions of ABAQUS were created. Similar to ANSYS, ABAQUS is a popular FEM software to be widely used for the simulation of complex solid mechanical systems. ABAQUS is usually used in tablet preparation simulation. In 1999, Femlab was published and has grown rapidly in the 21st century. Then, it was developed and renamed COMSOL. COMSOL Multiphysics is well known for handling in the multiphysics field, especially in microfluidic simulation of pharmaceutics. In 2015, EDEM software was released. EDEM is the first multi-purpose software that focuses on solving discrete-phase problems. EDEM is suitable for the simulation of granular agents. OpenForm was first used in a thesis in 1996. When using CFD to solve fluid problems, OpenFOAM and ANSYS Fluent are two commonly used simulation packages. OpenFOAM is an opensource solver library written entirely in the C++ language, and provides users with the possibility to flexibly customize the solver. ANSYS Fluent is a tool for CFD analysis developed by ANASYS, Inc. In addition, there are also coupling solvers such as CFDEMcoupling. CFDEMcoupling usually works in a coupled environment of OpenFOAM and EDEM. In this case, OpenFOAM handles the fluid simulation and EDEM handles the particle motion simulation. CFDEMcoupling and coupled CFD-DEM provide strong support for the calculation and analysis of complex systems. Furthermore, the PBM is often used to predict particle growth, nucleation and fragmentation. Commercial software such as ANSYS, MATLAB and EDEM all contain the PBM. In all of the above software, ANASYS, ABAQUS, COMSOL Multiphysics and EDEM are fee-based commercial software. OpenFOAM is an open-source software suite, and anyone can freely download, use and modify its published source code.

This paper systematically reviewed the current status of the application of mechanistic models in pharmaceutical preparation research. At present, numerical simulation has been widely adopted as an auxiliary research tool for granules, tablets, aerosols, sprays, powder aerosols, nano dosage and microneedle. However, for other unmentioned dosage forms, the application of numerical simulation is relatively rare or has not been reported yet. This article introduces the use of mechanistic modelling principles and methods based on previous studies. This review focuses on the brief principles of numerical techniques and their scope of application as well as specific application cases instead of theoretical foundations. This will be more applicable for pharmacy researchers to efficiently apply computer techniques across disciplines. In addition, this paper provides a thorough discussion of the limitations and common solutions of numerical simulation in the application of pharmaceutical formulations. A comprehensive summary of the chapter’s key literature is provided using a table, which is attached to the end of each chapter.

## 2. Application of Mechanism Models in Granule Investigation

As a commonly used traditional pharmaceutical dosage form, granules have many advantages such as rapid absorption, small dosage, and easy to take, carry and transport, thus they play a key role in pharmaceutical preparations. Many dosage forms have intermediates in granular form, such as capsules and tablets. Therefore, it is particularly critical to further analyze the process of granule preparation. Currently, the equipment is commonly used in the industry for the preparation of granules including spray granulators, fluidized bed granulators, and twin-screw extruder granulators. The internal mechanical geometry of these devices is complex and invisible, which is difficult to be directly observed during the actual granulation process. In addition, it is an extremely time- and resource-consuming task to explore suitable preparation conditions when a new active pharmaceutical ingredient (API) needs to be prepared as granules. Therefore, it is important to use numerical simulation methods to select and determine the preparation conditions to save time and costs.

In the numerical simulation of the preparation of granules, the main tasks focus on three key processes, drying, mixing, and granulation. Then, this paper will address these three aspects of the study in detail, respectively. Table 1 summarizes literatures related to numerical simulation mechanism modeling of granules at the end of this chapter.

### 2.1. The Drying Step

In the drying process, there are a variety of techniques, such as freeze drying, vacuum drying, and fluidized bed drying. Currently, there are several cases of successful application of numerical simulation for both fluidized bed drying and spray drying. Most of these simulations are based on CFD and combined with the DEM to simulate the distribution of particles at the same time. The coupled model of CFD and the DEM is commonly used to simulate multiphase flow scenarios containing fluids and solids, especially in the simulation of fluidized bed drying and spray drying. The model has the capability to accurately simulate the complexities of water vapor transport in air and water vapor transfer between particles and air, taking into account both mass and heat transfer processes.

**Figure 2:**
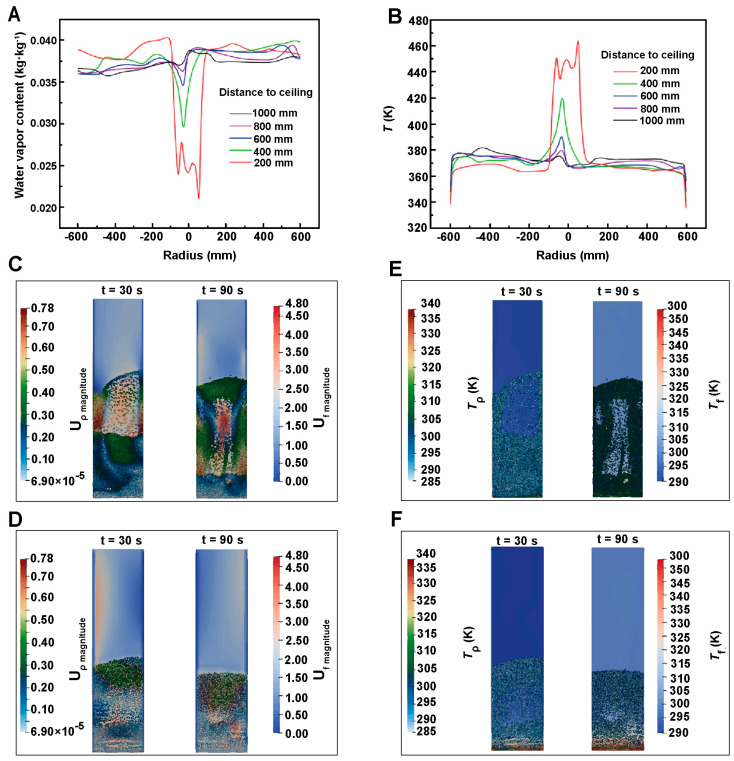
(**A**) Moisture distribution inside the drying chamber obtained from spray drying simulation. (**B**) Temperature distribution inside the drying chamber obtained from spray drying simulation [23]. (**C**–**F**) Particle velocity clouds of the original (**C**) and coarse (**D**) particle sizes at two time points in the fluidized bed; temperature clouds of the original (**E**) and coarse (**F**) particle sizes at two time points in the fluidized bed [24].

Spray drying simulation involves complex interactions of gas, liquid and solid phases, in which liquid droplets exist in air in discrete form [22]. Through numerical simulation, we can accurately predict the moisture content inside the drying chamber, the moisture distribution on the solid surface, and the temperature distribution. By using CFD, the evaporation and motion of droplets during spray drying have been thoroughly investigated by Zhang et al. [23]. In their simulations, the gas was considered as a continuous phase and described by the Eulerian method. However the droplets, as a dispersed phase, were simulated by the Lagrangian method. Meanwhile, they considered the effect of vortex motion on the fluid flow and chose the RNG k-ε turbulence model for numerical simulations. The numerical results showed that the temperature at the inlet was higher, while the temperature at the wall decreases dramatically, which was consistent with the experimental results. As shown in Figure 2A,B, the distribution of moisture content in the drying chamber showed an opposite trend to the temperature distribution. By turning on the particle tracking function, they used the Lagrangian model to calculate the motion trajectories of the discrete terms and the temperatures at each location, and then predict the moisture content at each location of the particle motion trajectories. During the whole drying process, they observed the air reflux phenomenon and the vortex motion of the droplets, and realized the visualization of the multiphysics field distribution and the trajectories of the discrete terms.

During the fluidized bed drying process, the rapid heat and mass transfer result in inhomogeneous physical properties at different place, and cause complex gas-solid two-phase flow. At present, the fluidized bed drying simulation mostly adopts the Euler model under the two-fluid model. This model establishes kinetic energy and continuity equations for the two fluids, and considers them as continuous phases coupled with each other [25,26]. In addition, the coupled CFD-DEM model is often used in fluidized bed drying simulations to obtain the properties such as particle size, water content, viscosity as well as the heat and mass transfer between the particles and the gas phase. However, the DEM has the limitation of the number of computational particles. Thus, many studies increase the particle size in the simulation or use a particle group to reducing the consumption of computational resources. Aziz et al. [24] investigated the feasibility of using a coarse-grained model for the simulation of a particle drying process under the coupled CFD-DEM modeling approach. As shown in Figure 2C–F, their results showed that the coarse-grained simulation coincided well with the original particle size simulation in terms of temperature results, but there were minor differences in velocity results. This research model provides new ideas and methods for the simulation of the fluidized bed drying process for large-scale particles.

### 2.2. The Mixing Step

DEM is the most commonly used model for numerical simulation of particle mixing. Although CFD has been used to simulate dense particle flows and gas-solid two-phase flows in particle mixing for more than fifteen years [27,28], the DEM has its own advantages. The internal algorithms the of DEM are constructed based on particle dynamics, which assumes that particles are regularly spherical, can undergo binary collisions, and are independently and randomly distributed in space [29,30]. The contact and cohesion forces between particles are main factors in the DEM, which applies Newton’s second law and the Lagrangian method. In order to perform the simulation, relevant parameters of the particles must be defined, such as density, viscosity, Young’s modulus, Poisson’s ratio, coefficient of restitution, and friction coefficient [31]. By using the DEM, we can obtain rich physical information in the particle mixing process such as path line plots, velocity vector plots and density distribution contour plots of the particles, as well as the specific position coordinate data of each particle. The above data provide an important basis for assessing the mixing quality.

**Figure 3:**
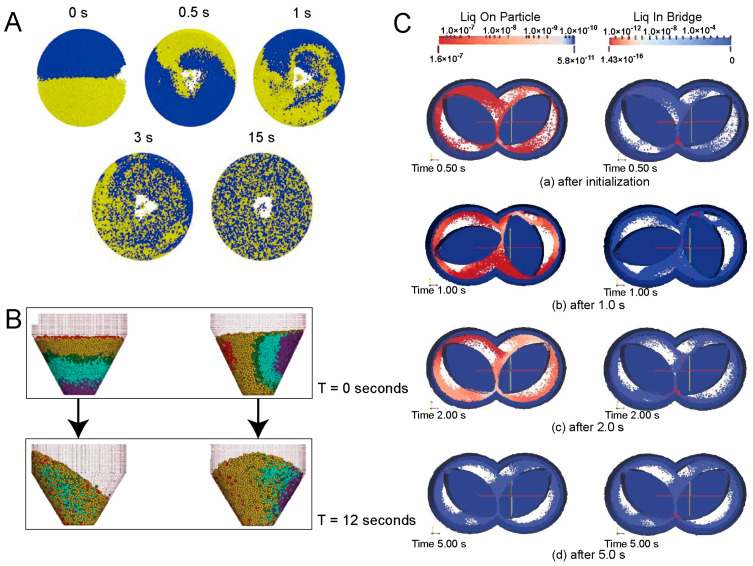
(**A**) Contour plots of particle distribution at each stage of mixing in the high-shear wet granulator [8]. (**B**) Mixing of tile-filled (left) and grid-like filled (right) particles of different properties at mixing times of 0 s and 12 s in the mixer [5]. (**C**) Distribution of moisture on the particle surface (left column) and in the liquid bridge (right column) at each stage of drying in the high-shear wet granulation process [33].

Tanabe et al. [32] established the simplified model by amplifying particle size and reducing agitator geometry. The simplified model used the DEM to quantitatively predict the probability density distribution of the API under a specific physical property and sampling scheme. The simplified model showed good accuracy in predicting the distribution of API. However, the accuracy of particle mixing in this scaled-size model was low. In addition, Tanabe provided a detailed description of the setup and operation of the numerical model as a valuable reference for similar studies. Furthermore, Fan et al. [8] investigated the mixing process in a high-shear wet granulator, focusing on evaluating the effects of density, particle size, and the volume fraction of each type of particles on the mixing homogeneity. They used different colored particles to mark the different properties of the particles and effectively observed the mixing at each stage through particle contour plots, as shown in Figure 3A. Further, Sen et al. [5] explored the process parameters on the mixing effect in the Bohle bin mixer, including particle filling pattern, filling percentage and rotational rate. The simulation generated contour plot of particle distribution showed that tiled filling had higher mixture uniformity compared to the vertical grid-like filling during the same mixture time. Meanwhile, the increase in stirrer speed also significantly accelerated the mixing process, as shown in Figure 3B. In summary, numerical simulation is an effective tool to provide key information on particle distribution, and investigates the effects of process parameters and material property parameters on particle mixing uniformity. These results provide important theoretical references for the selection of mixing parameters in industrialized production.

### 2.3. The Granulation Step

There are two kinds of particle granulation methods: dry granulation and wet granulation. Numerical simulation is mainly applied to the study of wet granulation, aiming to investigate the distribution of liquid and solid phases, the cohesion process of particles, and the prediction of particle growth. In addition, numerical simulation can also demonstrate the effects of different process parameters and raw material properties on particle cohesion and growth. Currently, the DEM, CFD and the PBM are commonly used in granulation process simulation. The DEM is widely used in fluidized bed granulation and shear granulation. The DEM usually adopts the Euler-Lagrange method, which reveals the global motion of the particle flow by calculating the trajectories of individual particles [34]. CFD is mainly used in the study of spray-drying granulation and can also be used in the simulation of fluidized bed granulation [35]. In the simulation of spray drying granulation, CFD can reveal the changes in momentum, energy, and heat of particles and fluids. In the simulation process, both Eulerian and Lagrangian models are used to study the laws of motion of the fluid. The Eulerian model studies the entire fluid as a mass. However, the Lagrangian model tracks each point. In the spray drying process, the Eulerian-Lagrangian model is often used to reveal fluid-particle interactions. The Eulerian model is used to clarify the drying process. The Eulerian model treats both the fluid and the particles as a continuum, and ignores the study of individual particles. The Lagrangian model focuses on the evolution of individual particles such as particle growth and mixing. Furthermore, the PBM is mainly used to simulate the nucleation, growth and aggregation of particles in the granulation process. During granulation simulation, the parameters need to be input including cohesion, friction, and coefficient of recovery between particles, resistance and friction between particles and walls, as well as surface tension between particles and liquids. These parameters are essential to simulate the particle granulation process.

#### 2.3.1. Application of the Discrete Element Model in Granulation

Daniel et al. [36] utilized the DEM to study multiple process parameters of the pelletizing process in a continuous twin-screw extruder, including screw configuration, rotational velocity, and feed rate. They evaluated the mixing homogeneity of the pelletizing process by calculating residence time. The “residence time” was defined as the average retention time of the micro-elements entering the continuous flow reactor at the same time. The results showed that axial mixing performed well with any screw configurations, while radial mixing performed well only with screws with 90-degree kneading elements. This study provides valuable guidance for the selection of screw configurations prior to experiment. Kumar et al. [33] focused on the simulation of liquid distribution, solid-liquid mixing, and kneading mechanisms during high-shear wet granulation. They paid more attention to the “liquid bridge” phenomenon formed in the presence of liquid on the surface of microscopic particles, which was caused by surface tension that interconnects the liquid between particles. The bridge forces played a key role in determining whether the particles were agglomerated or not. Kumar revealed in detail the formation of the bridges, the rupture process of the bridges, and the effect of the bridges on the interactions between the particles. All the above physical properties resulted in the agglomerative behavior of the particles. The contour plot in Figure 3C vividly demonstrated the rapid decrease in liquid content over time during particle drying. Figure 3C(a) demonstrated that most of the liquid on the surface of the particles was squeezed into the liquid bridge when the wet powder was pressurized between tightly engaged pinch disks. The results helped to elucidate the effect of liquid loading on the granulation process. In addition, Goldschmidt et al. [37] also used the DEM to investigate factors such as fluidization rate, spray rate and spray mode during spray granulation, as well as the agglomeration process of particles and liquid droplets. The results were in good agreement with the experimental data with an error of only 4%, thus verifying the validity and accuracy of the model.

#### 2.3.2. Application of Computational Fluid Dynamics in Granulation

**Figure 4:**
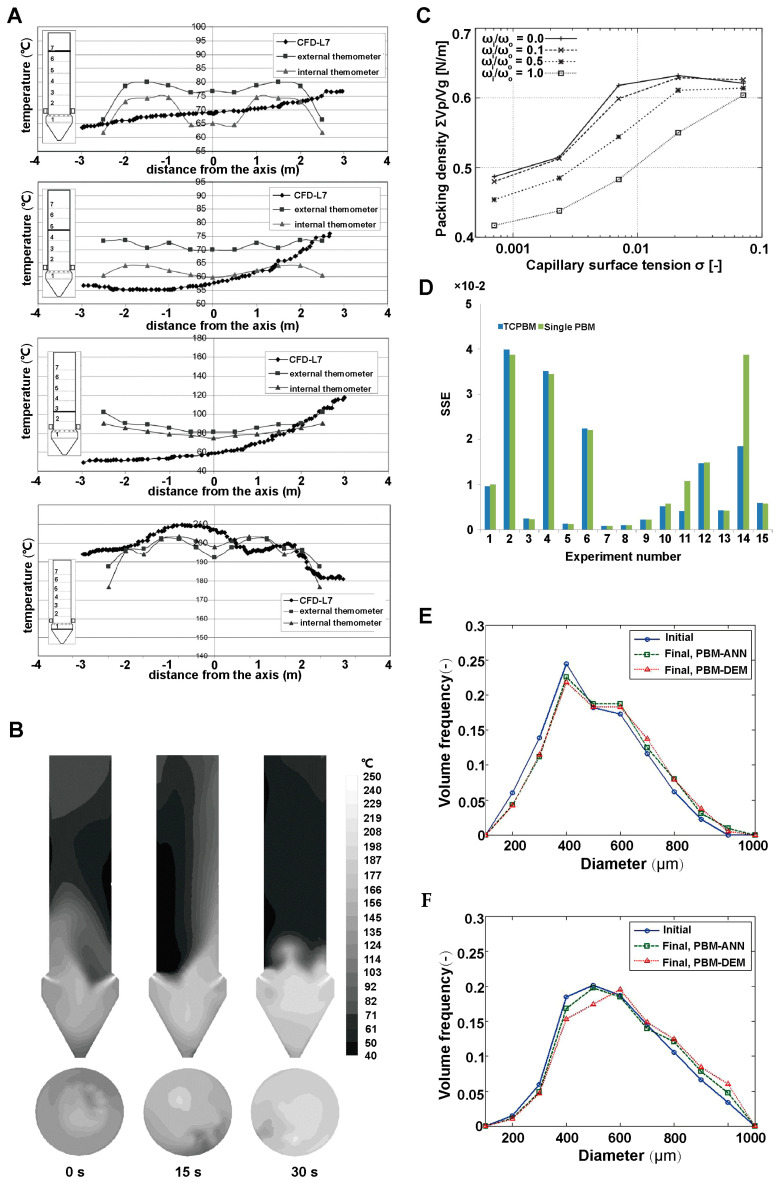
(**A**) Radial air temperature profiles (averaged in time) on dryer levels of 7/5/3/1. (**B**) Air temperature profiles in vertical and horizontal cross-sections of the dryer at consecutive time steps [38]. (**C**) Mean packing density of dried granules as a function of the capillary surface tension σ. [39] (**D**) Comparison of the sum square of error for each experiment of the TCPBM and the single PBM [40]. (**E**,**F**) Particle size distributions obtained from the PBM-ANN and PBM-DEM models over a 10 s interval for impeller speeds of (**E**) 60 RPM and (**F**) 90 RPM. Initial size distributions were obtained from the PBM-ANN models at t = 100 s [4].

CFD are used to simulate fluid motion by solving the energy conservation equations and mass conservation equations, and are widely used to study the motion of the gas phase and the flow of particle streams in the granulation process. CFD can predict the airflow behavior, particle behavior, temperature distribution and moisture distribution, which optimizes the processes and parameters to improves the granulation efficiency. Wawrzyniak et al. [38] constructed a CFD model for heat transfer in a countercurrent industrial spray dryer to calculate the temperature distributions at various locations in the drying tower. They obtained the temperature data from the model calculation and plotted a line graph with the height of the drying tower, as shown in Figure 4A, and generated a contour plot of the temperature distribution, as shown in Figure 4B. In Figure 4A,B we can observe the height instability of the airflow in the tower as well as the inhomogeneous distribution of the temperature on each cross-section. Based on these temperature distribution characteristics, they further established the power density distribution function F(x) to predict the evaporation power distribution at different locations in the tower. Further, the case of using CFD can only consider the global particles as fluid and cannot obtain the behavior of individual particles. Thus, some researchers use CFD-DEM coupling to take into account the behavior of both the airflow and the individual particles. Breinlinger et al. [39] used a coupled CFD-DEM model to investigate the effect of surface tension on particle morphology during spray drying granulation. They calculated the inter-particle surface tension and particle density and then plotted the surface tension-particle density function, as shown in Figure 4C. The plot showed strong positive correlation between particle density and surface tension. In addition, the density of particle clusters formed by particle agglomeration was also strongly correlated with the surface tension, and the finding provided an important basis for an in-depth understanding of the changes in particle morphology during spray-drying granulation.

#### 2.3.3. Application of the Population Balance Model in Granulation

The PBM is mainly applied to probe the detailed behavior of particles in the granulation process. It can simulate the processes of growth, nucleation, aggregation and fragmentation of individual particles, and predict particle size in the granulation process. Liu et al. [40] combined the two-compartmental population balance model (TCPBM) with CFD to investigate the particle size distribution and its evolution in the spray fluidized bed granulation process. The TCPBM performed well in predicting the dynamic changes in particle size under different binder solutions in the pulsed spray fluidized bed granulation process. By comparing 15 sets of simulation results, as shown in Figure 4D, the TCPBM was more accurate in predicting the average particle size with a smaller sum of squared error compared to the PBM. Furthermore, Barrasso et al. [4] used the DEM to train an artificial neural network (ANN) and the combined PBM with ANN in order to simulate the wet granulation process. In this process, they utilized the impeller rotational speed and collision frequency as key parameters to describe the particle size and distribution. As shown in Figure 4E,F, the PBM-ANN and the DEM-PBM showed a good consistency in simulating the particle size distribution within a 10 s interval at impeller speeds of 60 RPM and 90 RPM, respectively. However, if the simulation extended to 180 s, the DEM-PBM costed a couple of months to calculate and provided detailed particle size distribution results. In contrast, the PBM-ANN costed a few seconds to complete the calculations and demonstrated consistent trends of the particle size distribution. Thus, the efficiency of simulation was significantly improved.

**Table 1:** Summary of the literature related to numerical simulation mechanism modeling of granules.

	Mechanistic Model	Simulation Platform	Content of Research	The Role of Simulation	Research Focus	Reference
Review	-	-	Advances in numerical simulation of unit operations for tablet preparation	Numerical simulation of particle drying, mixing and granulation processes	Overview of numerical modelling	[31]
	DEM	-	Application of the discrete element method in modelling production processes in the pharmaceutical industry	Contact models and input parameters are essential elements in DEM simulation	Overview of DEM	[19]
	CFD-DEM	-	Prospects and limitations of CFD-DEM applications in process engineering	Current status of advances in coupled CFD-DEM simulation	Detailed description of the model	[41]
	PBM, CFD	-	An overview of mechanistic modelling of wet particle drying processes	Discuss the different steps in the modelling approach	Fluidized bed drying	[42]
Drying	CFD-DEM	OpenFOAM^®^	Numerical study of particle dynamics in a rotor granulator	Particle dynamics and particle contact behavior of fluidized bed rotor granulators obtained by simulation	Coating of particles	[43]
	CFD-DEM	CFDEM^®^ coupling code	Study of the drying process of wetted pharmaceutical particles in a fluidized bed dryer using a coupled CFD-DEM approach	The model predicts the drying rate as a function of inlet air velocity and inlet temperature, capturing the difference between wet and dry particle kinetics	Influence of process parameters on drying	[44]
	CFD-DEM	-	Application of a coarse-grained coupled CFD-DEM model to predict particle fluidized bed drying and heat transfer processes	The coarse-grained model was able to simulate the drying process of a large number of wet drug particles in a fluid bed dryer	Modelling improvements	[24]
	CFD-DEM	Open-source codes CFDEM	Numerical methods for particle-fluid thermal convection, spraying of coating fluids and evaporation of sprayed fluids on particle surfaces were investigated	The simulation results showed that there are three drying modes for the particles during the coating process	Mass and heat transfer	[45]
	CFD	ANSYS Fluent	Study of the effect of different operating conditions of dryers on drying	Finding optimal process conditions using numerical simulation	Process optimization	[46]
	CFD	-	Simulation of particle drying in a fluidized bed dryer using a two-fluid model coupling mass and heat transfer	Two-dimensional CFD simulations yielded a continuous drying profile. Three-dimensional CFD simulations yielded particle cross-section volume fractions with a core-annular structure	Mass and heat transfer	[47]
	CFD	-	Three-dimensional modelling of two-phase flow and transport in centrifugal spray dryers	Simulations were carried out to detect and analyze the temperature, humidity and velocity distributions of the hot air in the drying tower as well as the droplet trajectories	Process parameters	[23]
	VOF-FVM	Open-source code Basilisk	A sharp phase transition model based on the VOF method was proposed to solve boiling and evaporating flows	The phase transition process was investigated by constructing a numerical model to solve the boiling and evaporating flow	model building	[48]
	DPM-CFD	Ansys CFD 16.1	This study developed a DPM-CFD model describing the particle flow in an apparatus operating in the fast-circulating dilute spout-fluid bed regime	The study obtained the key device areas and key process parameters, as well as the factors affecting the accuracy of numerical simulation	Model building	[49]
Mixing	DEM	-	Effect of particle size and agitator size on particle mixing performance	DEM analyzed the effect on mixing of using large size particles and small size mixer models	Particle mixing performance	[32]
	DEM	-	Study of mixing homogeneity in a high shear wet granulator using DEM	Simulation to analyze the effect of particle size, density and volume fraction on mixing homogeneity	Material properties and mixing effect	[8]
	DEM-PBM	MATLAB	Capturing the dynamics of continuous powder mixing using a PBM model	Powder fluxes obtained from the DEM were input into the PBM to track key attributes such as API composition and relative standard deviation	Process parameters and powder mixing	[50]
	DEM	-	Experimentation and simulation of the mixing of multi-component particles	Numerical simulation results showed that radial mixing in the mixer is faster than axial mixing	Process parameters and mixing effect	[5]
	DEM	EDEM	An investigation on the predictive capabilities of DEM simulations of a powder mixing process in a laboratory-scale paddle blade mixer is presented	Evaluating the ability of viscoelastic-plastic friction-adhesion DEM contact models to predict the mixing process of zeolite powders with different water contents	Powder moisture content simulation	[51]
	DEM	-	The mixing behavior of particles in a rotating drum mixer was analyzed by experiment and DEM simulation	The effect of particle density on the mixing behavior was analyzed using DEM	Particle density	[52]
	CFD	ANSYS Fluent 2020 R1.	Using CFD to simulate powder fluidization in turbulent channels	Investigating the effects of packing limit, coefficient of recovery and turbulent diffusion on packing performance	Process parameters and mixing effect	[29]
	PBE	-	The study compared the cell average technique and finite volume scheme in terms of their accuracy and applicability in predicting the mixing state	Both numerical methods predicted the average size of particles well, but the finite volume scheme took less time	Models comparison	[53]
Granulation	DEM	EDEM	Simulation concept of high efficiency granulation in twin-screw granulator	Using cycle simulation, the results were similar to those of full-scale simulation, but the computational cost was significantly reduced	Simplification of the granulation model	[54]
	DEM	EDEM	Dry powder mixing in a continuous twin-screw extruder	DEM provided information on particle-scale mixing patterns within the extruder	Process parameters and mixing effect	[36]
	DEM	SALOME	Modelling particle size and understanding liquid distribution in twin-screw wet granulation	Time evolution of particle flow and inter-particle fluid distribution obtained by simulation	Process parameters and mixing effect	[33]
	DEM	EDEM 2.5	Reduced-order PBM-ANN model for DEM-PBM of wet granulation process	DEM tracked each particle individually, as well as modelling spatial variations in particles	Coupled DEM-PBM	[4]
	DEM	-	A new discrete element spray granulation model was proposed	Models captured key features of fluidized bed hydrodynamics, liquid-solid contact and agglomeration	Process parameters and particle growth	[37]
	CFD	Fluent 13.0	Analysis of a two-compartment population equilibrium model for pulsed spray fluidized bed granulation using CFD modelling	Details of the flow characteristics and distribution of particles between the wetting and drying chambers were obtained by simulation	Coupled CFD-PBM	[40]
	CFD	-	A 3D computational fluid dynamic modeling of atomization and fluidization of urea was simulated in an external mixing, bottom spray, fluidized bed granulator	Simultaneous solution of mass, heat and hydrodynamic equations to simulate a realistic urea fluidized bed granulation plant	Study of the atomization process	[35]
	CFD-DEM	OpenFOAM	Modelling the effect of surface tension on particle morphology during spray drying	Simulations revealed a clear non-linear relationship between high surface tension and dense particles	Effect of surface tension on granulation	[39]
	CFD-DEM	in-house DEM code, AVL-Fires	A new hybrid approach was proposed to solve the CFD-DEM problem in gas-solid fluidized bed systems using an efficient coupling method suitable for large-scale simulations	The model could perform massively parallel DEM simulations with millions of particles	Optimization of CFD-DEM models	[55]
	CFD-DEM-PBM	-	The mechanism of particle formation in seed granulation was elucidated and the suitable conditions for optimal granulation were predicted	Using the DEM to evaluate particles and CFD to study binder-fluid interactions, the CFD-DEM-PBM framework could link the effects of particle-fluid interactions	Granulation process monitoring	[34]
	PBM	-	A new approach was proposed for finding the analytical solution of population balances for aggregation and fragmentation process	The modelling approach avoided the problem of unstable solutions	Innovations in models	[56]
	PBE	-	Development of two approaches based on finite volume schemes for solving both one-dimensional and multidimensional non-linear simultaneous coagulation -fragmentation population balance equations	Models were sought for solving problems involving twin-screw granulation and spray fluidized bed granulation for industrial applications	Modelling exploration	[57]
Particle coating	DEM	DEM code	Three methods of modelling the coating process using the DEM were studied and compared	The key parameters of the spray model were found through the sensitivity of the model to the parameters	Explore the model	[58]
	CFD-DEM	-	Simulations investigated the dynamic interactions of particles, coating spray droplets and airflow throughout the coating system	Coupled CFD-DEM approach for modelling particle and gas flow to study factors affecting coating distribution	Assessment of coating indicators	[59]

## 3. Application of Mechanism Models in Tablet Investigation

Numerical simulation plays an important role in tablet compression and coating. For numerical modeling of tablets, there are two main numerical methodologies: the FEM and the DEM. On the one hand, the FEM simulates the powder as a continuous medium. In tableting preparation process, the FEM defines the material behavior by means of the constitutive model. The constitutive model describes the mechanical properties of the material by means of mathematical expressions, i.e., the relationship between stresses, strains, strengths, and time. In the constitutive model, the powder is considered as a continuous medium in order to study the mechanical behavior during the tableting process. On the other hand, the DEM reduces the powder to its original particle form for study. It focuses on the variation in physical properties at the particle level and probes deeply into various physical phenomena at the particle level during the tableting process. Therefore, the DEM has wide applications in powder mixing, powder transportation, film coating, tablet motion and granulation quality studies. Currently, the DEM is a capable methodology to investigate the variation in physical characteristics at the granule level [60]. However, the DEM mainly involves the computation of a large number of particles, achieving thousands of particles. Thus, a complete computational cycle may take a week or even longer. The DEM can provide accurate simulation results at the kilobit particle level, but it also requires higher time costs and computational resources. To overcome these limitations, the DEM is often used in conjunction with other models. For example, the FEM-DEM and the DEM-PBM are commonly used coupling models. Among them, the PBM is mainly used in tablets for particle tracking and describing the dynamics of the particle system, and the PBM is often coupled with the DEM to provide more comprehensive simulation and analysis. Through this combined application, the advantages of various models can be fully utilized, so that complex processes such as tablet compression and coating can be simulated and analyzed more accurately. 

Table 2 summarizes literatures related to numerical simulation mechanism modeling of tablets at the end of this chapter.

### 3.1. Application of Finite Element Method in Tablets

**Figure 5:**
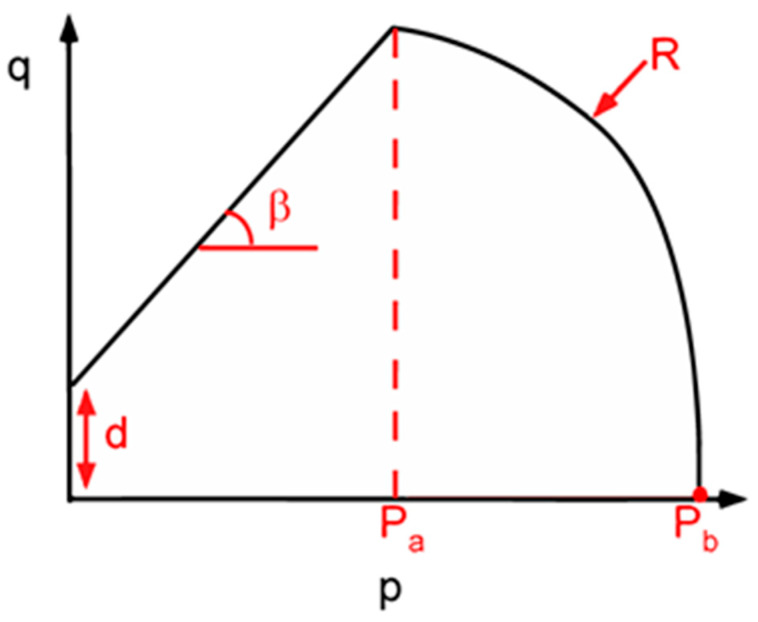
A schematic of the DPC yield surface showing the role of each parameter that describes the surface. The parameters are von Mises stress (q), hydrostatic stress (p), cohesion (d), the internal angle of friction (β), hydrostatic yield stress (Pb), evolution parameter (Pa), and the cap eccentricity parameter (R) [65].

The FEM treats the powder as a continuous medium and is mainly used to deeply investigate the relationship between stress and strain during the tableting process, the variation in each parameter with time, and the moisture distribution inside the tablets. By finely controlling the stress and strain during the whole tableting process, the simulation is able to predict potential forms of tablet breakage such as lobes and top cracks. In the literature on pharmaceutical tableting simulation, the constitutive model has been widely used for its ability to accurately describe the stress-strain behavior of materials. Initially developed in the field of geotechnics, the constitutive model has been successfully applied in the field of tablet manufacturing to provide comprehensive stress, strain, and breakage information for the tablet compression, decompression, and ejection processes. Currently, the commonly used constitutive models include the Drucker-Prager Cap (DPC) model [61], the Cam-Clay model [62], and the DiMaggio-Sandler model, which are all able to simulate the tablet compaction tightness well. Among them, the DPC model is the most popular. On the one hand, the DPC model is more accurate for the representation of the particle shear damage and plastic yielding process. The shear failure segment in the DPC model provides a criterion for the occurrence of shear flow, which is dependent upon the cohesion and the angle of friction of the granular materials according to the Mohr-Coulomb hypothesis. On the other hand, the DPC model can be characterized well with experiments on powders [63,64]. The DPC model includes five yield surface parameters, cohesion, the internal angle of friction, hydrostatic yield stress, evolution parameter and the cap eccentricity parameter; two elastic parameters, Young’s modulus and Poisson ratio, as shown in Figure 5.

**Figure 6:**
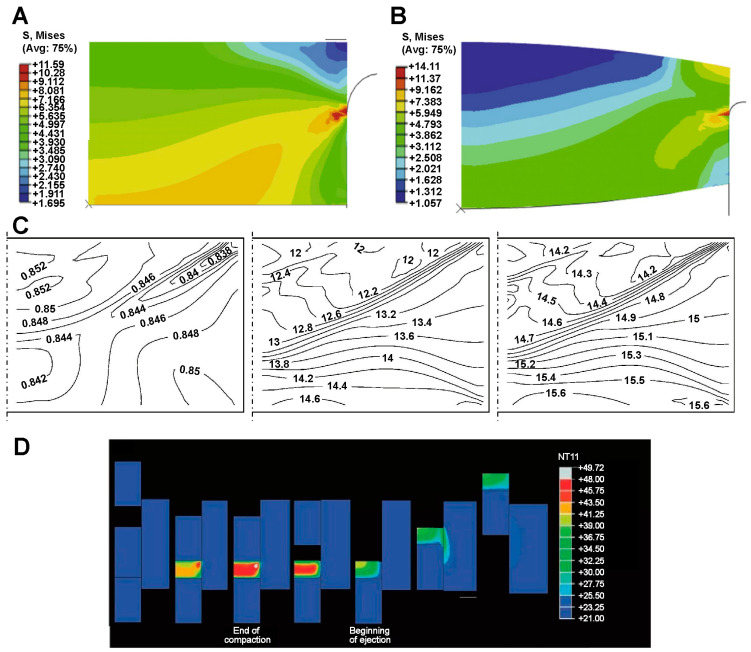
(**A**) Stress distribution of powders compacted by flat-face punches without wall friction during ejection. (**B**) Stress distribution during ejection using concave punches [68]. (**C**) Contour plots of the relative density (left), hydrostatic pressure (MPa, middle), and von Mises stress (MPa, right), showing the development of diagonal band characterized by low relative density and strong stress gradients during unloading of the tablet obtained for a minimum punch-to-punch distance of 3.9 mm [69]. (**D**) Temperature evolution during compaction and ejection sequence [71].

In the finite element simulation of tablet preparation, analyzing stress-strain and different formulation parameters can effectively predict potential regions of tablet breakage and their failure modes. Mazel et al. [66] used the DPC method to investigate the effects of shear stress, tensile stress, and the thickness of pressed powder on tablet capping and lamination. The simulation results showed that a decrease in tablet thickness leads to an increase in axial tensile stress. Biconvex tablets were more susceptible to lamination at high curvature and high residual die wall pressure. In addition, Gómez et al. [67] investigated the shear stresses generated within the tablets after compression using different excipients and confirmed that there was a direct relationship between shear and top cracking of the tablets. The material properties and the shape of the punch had a direct effect on the geometric properties of the tablets. The increase in the bevel angle of the punch induced the powder to move from the sides to the center during compaction. The phenomenon resulted in greater shear and risk of top cracking. Han et al. [68] explained tablet breakage by simulating the evolution of the powder density during tablet compaction. They used an improved density-dependent DPC model for numerical simulations. As shown in Figure 6A the planar punch tablet had a large stress concentration in the middle of the edge at the ejection, while the upper corner edge was a low stress zone with the tendency to edge spall. In contrast, as shown in Figure 6B, the concave punch tablet had a tendency to top crack at the ejection, because it has a low stress zone in the top and a high stress zone in the middle. It demonstrated that concave tablets were superior to flat tables in terms of edge spalling resistance, but the risk of top cracking was greater. This phenomenon may be caused by uneven density distribution due to punching geometry. The above researchers predicted tablet breakage through the density distribution. Further, Frenning’s model introduced hydrostatic pressure and von Mises stress gradient as early warning indicators of tablet top cracking [69]. The simulation observed a diagonal band with low relative density as well as strong hydrostatic pressure and von Mises gradients in the tablets, as shown in Figure 6C. These results coincided with the diagonal band shape top cracking reported in lactose tablets. It may indicate the risk of top cracking of the tablets. Generally, the FEM can effectively characterize the stress-strain changes caused by manufacturing process variables (e.g., excipient differences [65]) and determine potential tablet breakage regions based on the distribution of residual stress and density [70].

Pharmacodynamic failures induced by the tablet manufacturing process have been a key issue. Temperature elevation during pharmaceutical tableting has been of interest since 1958 [72]. Temperature elevation not only affects the physicochemical properties of pharmacodynamic ingredients (e.g., stability, crystalline state), but also may change the friction and excipient properties [70]. Zavalian et al. [71] predicted the transient temperature change during drug tablet compression by using the coupled thermal FEM and DPC model. The simulation results showed at the end of the tablet compression phase, a transient temperature peak occurred at the center of the tablet, in Figure 6D. Although the heat generated by the compression of individual tablets only led to a slight increase in temperature, the heat accumulated inside the tool during continuous production may result in a significant increase in temperature. This simulation results were in general agreement with the experimental results, indicating that the FEM can effectively monitor the transient temperature trends during the tablet pressing process.

Critical quality attributes of tablets include mechanical strength, disintegration, swelling, erosion and dissolution [2]. These critical quality attributes are strongly related to the dissolution rate of the API. Suitable tablet quality attributes often require extensive experiments, including the choice of excipients, API particle size, dose administered, and disintegrant position. The application of mechanistic models can speed up this optimization process. Kalný et al. [73] proposed a model based on the DEM for simulating the decomposition of ibuprofen tablets that contain APIs and disintegrants. The model was able to predict the increase in the average size of the segments as well as the profile of the distribution. The results showed good agreement with the experiment data. Kimber et al. [74] used the DEM combined with inter-particle mass transfer to simulate the radial swelling and dissolution of cylindrical tablets. Simulations showed the curves of tablet radius and API concentration over time. The results showed that diffusion coefficient and disentanglement threshold of the polymer were correlated with tablet dissolution. Hao et al. [75] used CFD to simulate drug release controlled by drug diffusion and polymer erosion. Simulations explored the effect of the position of tablets at the bottom of the container on its polymer erosion and drug release. Results showed that tablets located away from the center of the container deformed faster than those located in the center. Kimber et al. [76] used the DEM to explore the effect of tablet shape, polymer properties and shell thickness, and boundary conditions around the tablet on drug release curve and tablet evolution. The results showed that moderately solubilized polymers had the fastest drug release. The release rate of the drug is constrained by the low permeability of the boundary.

The QbD philosophy emphasizes overall consideration of design parameters, process operations and properties. It means that accurate design of preparation parameters is a guarantee of satisfactory quality. Another point is how different tablet shapes and properties of excipients effect on drug efficacy. Takayama et al. [77] used the DPC model to investigate the effects of the angle and depth of scoring on stress-strain to address the problem of poor uniformity between two parts of scored tablets. Their results showed that the stress on the scribe line reached the maximum value when the scribe angle was 45°. And the deeper scribe enables to achieve the homogeneity of the two halves. In addition, they analyzed the residual stresses in different formulations of tablets after applying pressure. It was found that the residual stress intensity was affected by the formulation composition, and the residual stress distribution was affected by the shape of the tablets. The difference in formulation composition was one of the important factors leading to the variation in the quality properties of tablets. By simulating the tableting process of different excipient formulations, it can provide a valuable reference for the selection of excipients in the pharmaceutical process. Numerical simulation has significant economic value in saving cost and speeding up the experimental process. Wang et al. [6] used the FDM to simulate the water-absorption property of substituting amylose and compared the simulation results with the experimental data to verify the accuracy of the numerical simulation. They further revealed the distribution of moisture in tablets, which was difficult to obtain under experiment conditions. Based on the moisture distribution, they also thoroughly investigated the diffusion kinetics of water in the substituted amylose. All the above studies provide important theoretical basis and practical guidance to optimizing the tablet preparation process and improving the quality of tablets.

### 3.2. Application of the Discrete Element Model in Tablets

There are functional differences between the DEM and the FEM in modeling the physical properties of powders during the compacting process. While the FEM can simulate the compaction of powders, it uses a linear model that may fail to simulate behaviors such as particle overlap at high strains [78]. In contrast, the DEM simulates a large number of particles using Newton’s second law and accurately represents the plastic deformation of particles using the plastic-contact law [79,80]. However, the DEM leads to a computationally intensive and time-consuming simulation process with high CPU storage space requirements. To solve the problem, researchers often use the DEM coupled FEM or the PBM to reduce time cost [4,50,81]. In the tableting process, the stress changes are affected by the homogeneity of mixing and the proportion of powders with different particle sizes. The uneven stress distribution may increase the potential possibility of breakage. Sen et al. [50] combined the DEM with the PBM to investigate the effects of key design and process parameters on the mixing effect. By using the DEM, they obtained parameters of powder fluxes. Then, they input the obtained parameters into the PBM to analyze key parameters such as API composition, relative standard deviation, and residence time distribution. The results showed that particle aggregation is produced due to particle cohesion. The results indicated that the combination of the DEM and the PBM could well characterize the dynamic process of powder mixing and reveal the relationship between powder mixing and key properties.

**Figure 7:**
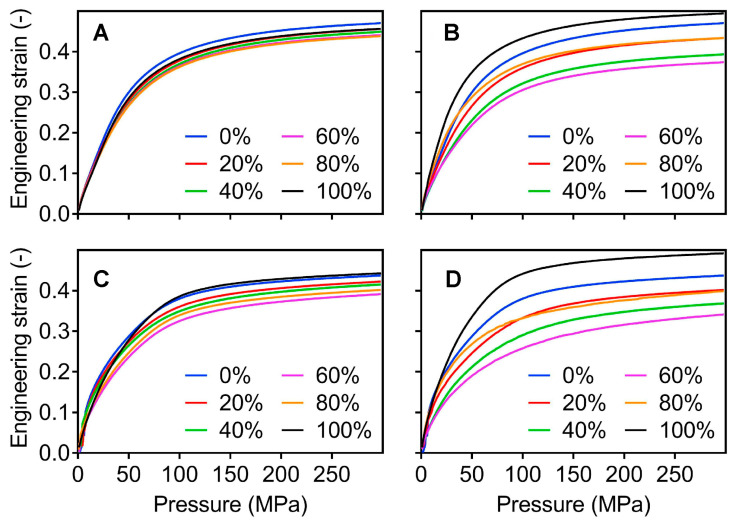
Typical compression profiles obtained from experiments (top row, panels (**A**,**B**) and simulations (bottom row, panels (**C**,**D**)) for the 1:2 (left column, panel (**A**,**C**) and 1:4 (right column, panel (**B**,**D**) size ratios (color online). Percentages in the figure indicate large particle content [83].

At the tableting process, the DEM is able to capture the changes in physical properties at the particle level and visually represent the states of rearrangement, elastic deformation, plastic deformation, and fragmentation of the particles during force application [82]. Nordström et al. [83] used the DEM to investigate the compressibility and tableting ability of bimodal particle mixtures formed from microcrystalline cellulose. They performed numerical simulations using the multi-body contact law, which explained the contact dependence caused by plastic incompressibility/geometrical hardening. The experiment results were in good agreement with the simulation results, indicating that particles with a size ratio of 1:4 had poor compressibility, as shown in Figure 7. Garner et al. [84] also used the DEM to simulate the mechanical behavior of the powder compaction process. They used a new plastic-contact law to represent the displacement behavior of particles in a highly cohesive environment and used a central composite design method to determine the response surface for model calibration. The results expressed that the decrease in residual die wall pressure and the increase deformation resilience in tablet after compression at low cohesion levels. The phenomenon was related to the elastic properties of the particles and decohesion of particle-particle contacts. In summary, the DEM successfully predicts and captures the kinetic behavior of powders during compaction in many previous studies, providing a scientific explanation of the mechanical properties for tablet quality issues.

**Table 2:** Summary of literature related to numerical simulation mechanism modeling of tablets.

Mechanistic Model	Conditional Model	Simulation Platform	Content of Research	The Role of Simulation	Research Focus	Reference
FDM	-	MATLAB^®^V.6	Diffusion kinetics of water in substituted rectilinear starch	Simulation to obtain the moisture fraction and moisture distribution of tablets with different tablet sizes and excipients	Moisture diffusion in the tablets	[6]
FEM	DPC	-	Stress-strain generated within tablets after diameter compression	Effect of indentation depth and tablet formulation on quality attributes of indented tablets	Scoring sheet process parameters	[77]
	DPC	ABAQUS^®^ Standard 6.13	The cracking mechanism of biconvex tablets and the effect of tablet thickness and pressing pressure on lamination were investigated	Increased compression pressure and reduced tablet thickness increase the risk of lamination	Biconvex failure factors	[66]
	DPC	ABAQUS^®^	The influence of powder properties and process parameters on the evolution of mechanical properties during tableting was investigated	Simulation identifies potential areas where tablet breakage may occur	Tablet breakage probe	[67]
	DPC	ABAQUS	Simulation of the mechanical behavior of microcrystalline cellulose drug powders during tableting	Introduction to the application of numerical modelling and parameterization	Introduction to the DPC model	[85]
	Improved DPC	ABAQUS2019	Developed and validated an automated parametric workflow to predict tableting of powder mixtures	Placing softer materials closer to the mould wall reduces residual radial stresses and reduces the risk of tablet breakage	Modelling improvements	[86]
	DPC	ABAQUS	Evaluated the effect of elastic parameters on numerical simulation results	Fluctuations in Young’s modulus and incorrect estimation of Poisson’s ratio could cause simulation errors	DPC model calibration	[87]
	DPC	ABAQUS	Investigated the pharmaceutical powder behavior during compaction	Simulations to obtain stress and density distributions in tablets and reveal the mode of tablet cracking	Tablet failure mechanisms	[63]
	Slightly modified DPC	ABAQUS	Study of single-end compaction of lactose powder to determine density and stress distribution within tablets and predict tablet failure mechanisms	The simulations illustrated the elastic and plastic deformation stages of the tablet pressing process	Pressing and unloading process	[69]
	DPC	ABAQUS/STANDARD	Presented a numerical approach to the prediction of temperature evolution in tablet compaction	Better prediction of temperature changes during tablet compression for tablets containing temperature-sensitive ingredients	Temperature variation during tablet pressing	[71]
	DPC	ABAQUS/Standard	The full pressurization of the simulated drug powder was carried out as	Simulation predicted edge shedding resistance and capping risk for convex and planar tablets	Compaction stress distribution	[68]
	DPC	-	Highlighting the potential and feasibility of FEM implementation in pharmaceutical tableting	The FEM and DPC could generate key information about the complex physical phenomena that occur during the tablet pressing process	Review	[70]
	DPC	ABAQUS^®^	Effect of friction between powder and tooling on the die-wall pressure evolution during tableting	Friction between powder and mould affected mould wall pressure	Effect of friction on compression of tablets	[88]
	DPC	ABAQUS	Mechanisms of crack formation in die compacted powders during unloading and ejection	Simulation elucidated a two-step mechanism of damage during tablet compaction generation	Crack formation mechanism	[89]
	-	ABAQUS	Study provided new information on how powder compaction behavior was influenced by particle size and particle shell-thickness	Investigating the effect of particle size and shell thickness ratio on the behavior of compacts	Particle compaction microscopic study	[78]
DEM	Multi-Contact DEM	-	Study of micro-particle behavior in the tablet pressing process and prediction of macro-behavior by micro-behavior	Compaction curves of microcrystalline cellulose stress-strain predicted by simulation	Micro-powder compaction	[60]
	-	-	Different approaches in the literature over the last 25 years were reviewed and parameter calibration is discussed	Guidance on the selection of appropriate model calibration methods and procedures	Calibration of the DEM	[90]
	-	EDEM 2.3	Experimentally and simulation-corrected parameters were entered into the DEM for simulation of tablet coatings	Input parameters relying on experimental data enabled a more accurate modeling	Determination of coating simulation parameters	[91]
	asymptotic limit models	MATLAB	Investigated intra-tablet coating thickness variability. The first attempt to directly compare experimental and simulate coating thickness distributions	Asymptotic values of changes in tablet internal coating derived from the new algorithm correlated well with tablet sphericity	Tablet coating simulation	[92]
	-	EDEM 2.4.4	Studied how operational conditions influence the coating uniformity	The simulation results helped to optimize variations in coating quality and minimize process time	Uniformity of tablet coating quality	[93]
	Kawakita equation	-	Studied compressibility and tablet forming ability of bimodal mixtures of differently sized granules	DEM was well suited at high relative densities when a suitable multi-particle contact model was used	Compaction of uneven particle sizes	[83]
	Edinburgh contact model	EDEM^®^ 2.7	Assessed the extent of particle rearrangement and the degree of anisotropy for uni-axial compaction of microcrystalline cellulose	Simulations were performed to obtain tablet loading curves and compared with experimental results	Particle rearrangement in tableting process	[82]
	novel contact model	-	The mechanical behavior of powders during die compaction was investigated	Simulations explored residual wall stress and rebound at the end of tablet compaction	die compaction of powders	[84]
	DPC&Bonded Particle Mode (BPM)	EDEM	Analyzed the microscopic mechanisms of the potato starch compaction and the origins of their breakage strength	BPM models could describe brittle, semibrittle or ductile fracture patterns of tablets	Tablet compaction mechanism	[94]
FEM, DEM	-	-	Verification of the feasibility of a finite/discrete element program to simulate compressed 3D particle components	Simulation was reasonable and effective for hyperelastic particle compression	Improvements in simulation methods	[95]
-	DPC	Medelpharm	Evaluation of the compaction characteristics of 14 different materials by simulation	Correlations between different pressing behaviors and simulation parameters were obtained	Model parameter	[65]

## 4. Application of Mechanism Models in the Study of Aerosols, Sprays and Powder Aerosols

**Figure 8:**
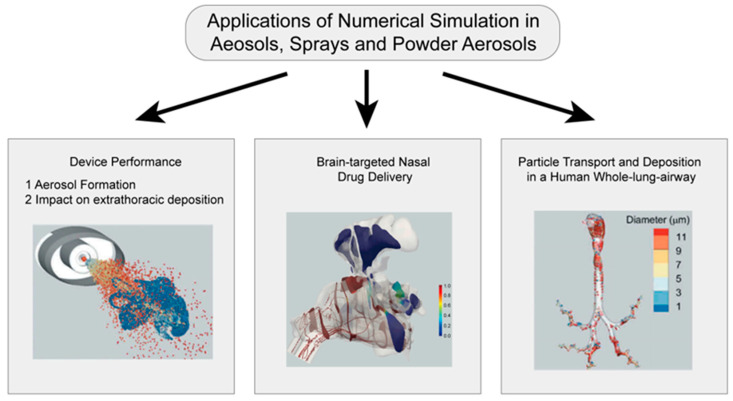
Applications of numerical simulation in aerosols, sprays and powder aerosols [96].

Numerical simulation plays an important role in the study of aerosols, sprays, and powder aerosols. The application scenarios of the simulation can be summarized in the following three aspects, as shown in Figure 8: (i) the simulation and evaluation of nebulization under airway drug delivery; (ii) the study of nasal-brain-targeted drug delivery; (iii) the analysis of whole-lung particle transport and deposition. The simulation of nebulizers mainly focuses on inhalers. This section will focus on methods of the establishment of geometry models based on physiological structures, and selection of suitable physical models. We also review numerical simulations of gas atomization, gas flow, particle deposition, and gas-particle-mucosal interaction processes. Researchers can adjust parameters of drug delivery based on simulation results to improve the drug deposition rate at the target location and optimize the design of nebulizers. Table 3 summarizes literatures related to numerical simulation mechanism modeling of aerosols, sprays, and powder aerosols at the end of this chapter.

CFD and the DEM are commonly used in the field of numerical simulation of aerosols, sprays, and powder aerosols. Both methodologies play a key role in analyzing the simulation of the motion of gases and particles in the airway, providing a thorough understanding of transport and deposition processes. CFD is able to calculate key parameters such as the velocity field and the pressure field of gases through the relevant transport governing equations [96,97]. We need to consider the conservation of mass, the conservation of momentum, and the effects of turbulence and swirling when using CFD models [98,99]. However, there are also several challenges in the application of CFD such as hygroscopic growth of particles in a humid respiratory tract, the localized supersonic flow of the gas, and the intermittent gas turbulence [100,101,102]. CFD is often used in conjunction with the discrete phase model (DPM) when performing respiratory tract simulations. DPM is a module in Fluent software that can be simplified to a discrete term model that does not take into account particle collisions and particle volumes. Compared with DPM, the DEM focuses on modeling the state of motion of particles, including particle movement, deposition, and particle-wall-gas interactions. By using the DEM, we can track the trajectory of individual particles and obtain their motion trajectories as well as the coordinates of the particles at any time. The DEM requires higher accuracy and much smaller time steps than CFD, thus consuming more computer memory and computational time. In order to study the particle-fluid interactions more comprehensively, we can adopt a coupled CFD and the DEM. Tong et al. [103] used CFD-DEM to simulate the agglomeration behavior of particles of different sizes in eddies and found that the dispersion of the powder was mainly affected by the particle-wall interactions. In the following study, they explored the powder de-agglomeration process because of the collision along the throat wall. The results showed that the agglomerates were damaged or fragmented for the first time after the collision, then the second collision produced more fine particles [104]. The above studies not only reveal the complex behavior of particles in fluids, but also provide strong support for scientific research and applications in pharmaceutics.

### 4.1. Inhaler Performance

CFD and the DEM are widely used in the modeling and simulation of dry powder inhalers (DPIs) with the main goal to predict the atomization process. The coupled models aim to optimize the design and achieve the maximum atomization efficiency under a certain energy. DPIs undergo complex processes during the formation of the aerosol such as initial fluidization within the powder bed, de-agglomeration due to collision, and de-agglomeration under the effect of airflow shear stress [105]. Due to the narrow geometry of the inhaler and the complexity of the internal airflow, researchers should take into account the turbulence of the airflow, particle-particle and particle-wall interactions such as collisions, electrostatic forces, and van der Waals forces [106,107]. Researchers commonly use Reynolds-averaged Navier-Stokes Equations (RANS) and Large Eddy Simulation (LES) to calculate turbulence [108,109]. Fluidization of drug API and carrier particles in the inhaler is driven by the fluid through resistance. The particle density determines the unidirectional or bidirectional coupling of particles and fluid. The drag force on a single particle is related to factors such as Reynolds number, volume fraction, and velocity deficiency between phases [110,111,112,113]. Inter-particle interactions are dominated by Van der Waals forces at short distances and electrostatic forces are more influential at long distances.

**Figure 9:**
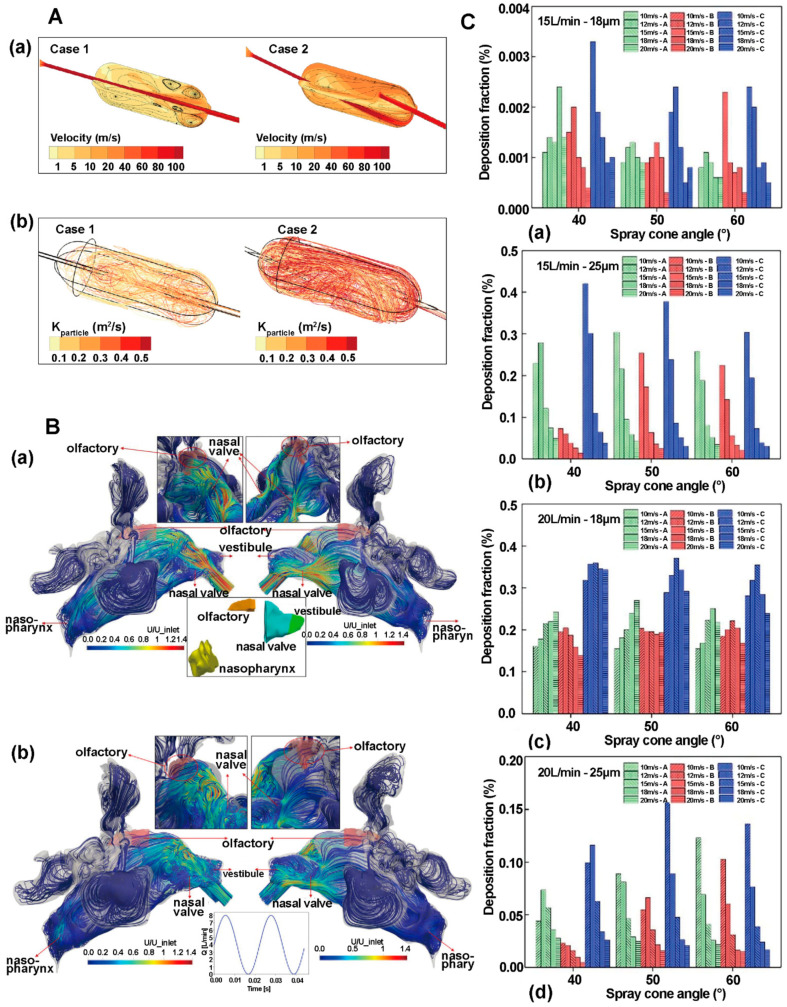
**(A**) Designs include Case 1, Case 2. (**a**) Contours of velocity magnitude on two axial planes and 2D stream traces on each plane. The central core of high-speed flow (inlet jet) induces significant secondary motion and complex vortical flow fields. (**b**) Particle trajectories contoured based on pathline integrations of exposure [100]. (**B**) (**a**) Streamlines of the normal(-ized) air velocity U in the nasal cavity for non-pulsatile (constant) inlet flow of 4 L/min at the end of inhalation period (t = 1.98 s). Right (inlet) and left (exit) views and close-up views of the olfactory region (shaded in red) are given in the left and right panel, respectively. In the bottom panel, different regions of the nasal anatomy are shown. (**b**) Streamlines of the normal(-ized) air velocity U in the nasal cavity for pulsatile inlet flow of 4 L/min with a frequency of 45 Hz (oscillation period T = 22.2 ms) at T/2 of the last modeled pulse (t = 1.98 s), where T/2 (middle of cycle of air flow pulsation) represents the condition where the inlet flow is equal to the average flow rate of the pulsatile flow profile. Again, the olfactory epithelium region is shaded in red [143]. (**C**) The deposition fraction of drug particles with different particle sizes in the OR under different delivery conditions at 15 L/min (**a**,**b**) or 20 L/min (**c**,**d**) auxiliary airflow. (**a**,**c**) Particle size is 18 um. (**b**,**d**) Particle size is 25 um [138].

In recent years, many simulations have appeared in the literature aiming to gain insight into the processes of fluidization, atomization and deposition of particles in inhalers, which come up frequently in reviews [114,115,116,117,118,119]. Numerical simulations of inhalers are mainly applied to the evaluation of existing inhalers [118,120,121,122], the development of new inhalers [100,123,124,125], and deposition-guided inhaler design [126,127,128,129]. For example, Longest and Farkas designed a novel air-jet DPI for pulmonary drug delivery [100]. They predicted both flow field-based and particle-based dispersion parameters that captured different measures of turbulence, as shown in Figure 9A. The results revealed a common paradoxical problem in inhaler design: increasing turbulence increased the device average emitted dose but decreased lung deposition. Relevant case support was provided by other studies by Longest et al. [96,130,131]. During capsule dry powder inhaler administration, the amount of drug depends on the design of the inhaler, the drug formulation and the inhalation operation. The inhalation operation affects both the airflow in the inhaler and the movement of the capsule [132]. Researchers used CFD-DEM modelling to explore the relationship among device design, drug formulation, inhalation process and the inhalation volume of capsule-based dry powder inhalers. CFD simulates the gas flow and particle flow. The DEM is used to simulate the trajectory and physical properties of particles. Firstly, dry powder inhalers (DPIs) need to achieve depolymerization of particles to form aerosols with a high fine particle fraction. The properties of the aerosol directly influence the site of drug deposition in the respiratory tract and further influence the drug efficacy. Longest et al. [133] used in vitro experiments combined with CFD to analyze the aerodynamic factors that play a major role in the formation of micron and submicron aerosols from the depolymerization of carrierless powders on capsule platforms. The results showed that turbulence was the main depolymerization mechanism. The best depolymerization effect was achieved when the median particle diameter of the drug powder was <1 μm. The results gave a reference for the selection of the powder size of the dry powder inhaler. Almeida et al. [134] evaluated the effects of drug/excipient bonding and DPI resistance on aerosol forming using CFD-DEM. The simulation showed that the pattern of powder dispersion was correlated with the resistance of the device. Secondly, the main factors that affect the amount of drug inhaled includes the flow field inside the capsule-based DPI, the inhaler design, the position of the capsule opening and the user’s inhalation angle. Shur et al. [135] calculated the flow field, pressure drop and carrier particle trajectories within the Cyclohaler and HandiHaler using CFD. The results were used to identify critical device properties and to design improved versions of the Cyclohaler. Zhu et al. [136] used CFD to study the effect of hole size of capsule openings on powder dispersion in atomizer device. The results showed that the capsule hole size affects the collision frequency and collision energy of the particles. Benque et al. [137] used CFD-DEM to study the effect of capsule position and capsule hole position on the discharge and dispersion of powder from a dry powder inhaler. They found that a constant airflow set in the simulation would lead to inaccurate results. Benque et al. [132] found that using the flow rate profile instead of a constant flow rate throughout the simulation provided a clearer view of the motion of carrier particles in the device.

In summary, the application of CFD and DEM in DPI studies is important to optimizing the design and improving the efficiency of nebulization. By accurately simulating the nebulization process and taking into account various complex factors, we can better understand and improve the performance of DPIs.

### 4.2. Nasal-Brain-Targeted Drug Delivery

Nasal brain-targeted drug delivery is a method of using the olfactory region of the nasal cavity to achieve brain drug delivery. Nasal brain-targeted drug delivery has the advantages of being able to bypass the blood-brain barrier and deliver drugs directly to the central nervous system. In order to study this process, researchers usually need to build a three-dimensional model of the nasal cavity in advance. The modeling involves performing a high-resolution computed tomography (CT) scan of the human head, segmenting and 3D reconstructing the CT image, and then importing the reconstructed model into the commercial software Geomagic for optimization [97,138,139,140]. The main variables include drug release mode [104,123,141], airflow characteristics [142,143], particle motion [103,138], and the effect of physiological or pathological changes on nasal drug delivery [144,145,146], and should be explored in the simulation. The variables of drug release include the angle of the nozzle in the nasal vestibule and the drug ejection velocity. The variables of gas flow characteristics include the linear velocity of the gas, and the flow pattern. Particle motion is influenced by factors such as particle size, inertial forces and gravity.

Farnoud et al. [143] used a patient-specific shaped nasal cavity model and analyzed the residence time of gases in the olfactory region for both non-pulsatile flow and bidirectional pulsatile flow. Under non-pulsatile flow, they observed a downward-moving cyclonic flow formed in the right nasal cavity after passing through the nasal valves, and part of the flow passing through the olfactory region at a high velocity. A homogeneous flow pattern was observed in the left nasal cavity, and the velocity of the gas flow decreased when reaching the olfactory region, as shown in Figure 9B(a). Under bidirectional pulsatile airflow, we observed complex flow forms in both the right and left nasal vestibules. The pulsation weakened the impact of the airflow on the roof of the nasal cavity, leading to a significant decrease in the flow velocity in both left and right olfactory zones, as shown in Figure 9B(b). The results showed that bidirectional pulse flow contributed to the drug deposition in the olfactory region. Ren et al. [138] simulated the fraction of drug deposition in the olfactory region (OR) under different auxiliary airflows, spray cone angles, particle sizes, drug particle delivery velocities, and ejection positions using a CFD method. They found that, under the same conditions, the fraction of drug deposition in the OR increased with the increase in particle size and decreased with the increase in delivery speed. Ren also found 40° spray cone angle was more favorable for OR deposition, as shown in Figure 9C. When auxiliary airflow was added, as shown in Figure 9C(c,d), the effect of particle size on deposition showed a trend that OR deposition increased and then decreased with the increase in particle size. These results provide an important basis for the design and optimization of nasal-brain-targeted drug delivery.

### 4.3. Whole-Lung Delivery and Deposition of Drugs

Pulmonary drug delivery is a method of delivering drugs in aerosol form to the lungs for pulmonary or systemic therapy. This therapy is facing tough challenges recently. To ensure the drug delivery and absorption in lungs, the liquid and the powder should be broken down into droplets or particles that are homogeneous and small enough to escape the barrier of the external thoracic airway. High-shear turbulence in the fluid can drastically reduce particle size. However, turbulence in the aerosols can lead to significant drug loss in the inhaler and external thoracic airway [147,148].

CFD has more advantages than the semiempirical model and one-dimensional whole-lung models in pulmonary drug delivery studies. CFD can reveal complex phenomenon such as transient flow, turbulence, particle dispersion, fluid-wall interactions, and size changes in hygroscopic particle. In airway simulation, the application of CFD can be summarized in two categories: partial airway simulation [149,150,151,152] and full airway simulation [153,154,155,156]. The establishment of full airway model needs to take into account the airflow diversion from the mouth, nose, throat to the lungs, and the decrease in Reynolds number due to the change in airway diameter, as well as the difference of physical properties of the walls in different physiological regions [96]. To deal with the challenges in whole-airway modeling, most researchers use the stochastic individual path (SIP) method [157,158]. The SIP method is based on physiological bifurcation data from anatomical studies and divided the airway in three separate parts to simulate [159,160], thus overcoming the problems of the length scale of airway and flow distribution variations.

**Figure 10:**
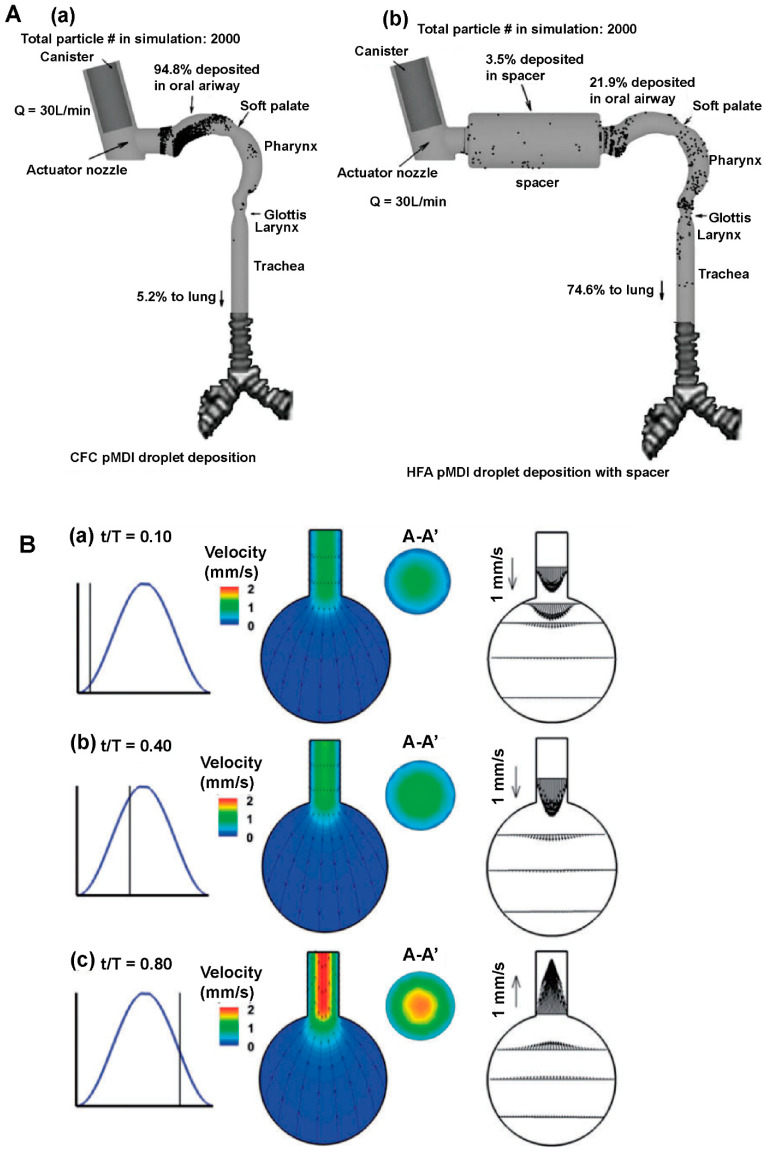
(**A**) (**a**) Simulation of respiratory and lung particle deposition after pressurized metered dose inhaler (pMDI) administration using chlorofluorocarbon (CFC) as propellant before inhaler modification. (**b**) Simulation of respiratory and lung particle deposition after pMDI administration using HFA as propellant with spacers [161]. (**B)** Airflow and vortex topologies inside the alveolus are shown at three different instants(**a**–**c**) within one breathing cycle [162].

Kleinstreuer et al. [161], for the first time, investigated methods to control aerosol transport in the lungs using CFD and simulated drug deposition in the mouth-throat region by using pressurized metered-dose inhaler. They effectively reduced the mouth-throat deposition and increased the lung deposition rate by using a new propellant, reducing the aperture of nozzle exit, and adding a spacer, as shown in Figure 10A. In addition, Xi et al. [162] constructed an idealized blind-end terminal alveolus model, as shown in Figure 10B. The model was used for quantifying alveolar particle deposition and evaluating the correlation between alveolar delivery efficiency and inhaled toxicant dosimetry. The results showed that alveolar deposition of aerosolized aerosols is highly sensitive to breath-hold time, particle release time, and alveolar size. Kolanjiyil et al. explored an alternative modeling to simulate the respiratory process by constructing an approximate whole-lung geometry model and implementing physiological boundary conditions. Their numerical results showed that the total particle deposition and local particle deposition results of the model agreed with the results of experiments, providing new ideas and methods for lung modeling. In summary, CFD simulation plays an important role in lung drug delivery, which not only contributes to a thorough understanding of drug transport and deposition mechanisms in the respiratory tract, but also provides a guidance to optimizing the design of drug delivery devices and dose regimen.

**Table 3:** Summary of the literature related to numerical simulation mechanism modeling of aerosols, sprays, AND powder aerosols.

	Mechanistic Model	Simulation Platform	Content of Research	The Role of Simulation	Research Focus	Reference
Review	CFD, DPM	-	A review examined different computational and experimental models for the direct delivery of therapeutic agents or colloidal carriers from the nasal cavity to brain tissue		Nasal-brain-targeted drug delivery	[163]
	CFD	-	An overview of the development of modelling techniques was given, and then the physiologically and clinically relevant findings arising from computational models were discussed		Clinical applications of model	[139]
	CFD	-	The aim of the review was to provide a more comprehensive and specific description of recent advances in the assessment of nasal drug delivery by in vitro and computerized methods. The article also detailed the CFD model		Nasal olfactory region deposition	[164]
	CFD	-	This paper provided a review of the development of CFD in the studies of nasal airway fluid dynamics, particle and filtering properties in health and disease.		CFD Applications	[97]
	CFD	-	This article reviewed the recent application of computational modelling to understand the nasal structure-function relationship following surgery in patients with sinusitis and nasal obstruction.		Evaluation of postoperative nasal function	[165]
	CFD	-	This review discussed the application of computational models to simulate the transport and deposition of inhaled pharmaceutical aerosols from the site of particle or droplet formation to deposition within the respiratory tract.		Whole-lung model	[131]
	CFD	-	This review article focused on CFD-based deposition modeling applied to pharmaceutical aerosols. Areas covered included the development of new complete-airway CFD deposition models and the application of these models on drug delivery		Whole-lung model	[96]
	CFD, DEM	-	This review article highlighted the development of methods for modeling flow and particle behavior in DPI devices and their applications, as well as summarized and critically evaluated recent simulation studies for different devices.		DPI numerical models	[115]
	CFD, DEM	-	The article reviewed the physical processes that contribute to DPI atomization and subsequent dispersion and deposition, and described how various length and time scales can be calculated by simulating the physical processes		DPI numerical models	[105]
Inhaler performance	CFD	Fluent 6.3.26	In this study, hollow cone and full cone spray patterns were evaluated to assess spray patterns that produced greater deposition in the mid-nasal region	Contours and streamlines of the flow field revealed that the presence of a spray device in the nasal vestibule produced higher levels of disturbed flow; CFD methods were demonstrated	Inhaler design on nasal deposition	[140]
	CFD	A	The effect of nasal drug delivery device and nozzle orientation on the deposition of spray droplets in the nasal cavity of a real human was investigated	Nasal spray ability was evaluated by simulation, taking into account respiratory conditions, direction of spray, droplet size, etc.	Spray performance evaluation	[104]
	CFD	ANSYS	Exploring different internal flow pathways in the patient interface region of a novel jet DPI to minimize device and extra-thoracic aerosol deposition losses.	Spray flow rates and deposition rates in various regions of the respiratory tract, as well as factors related to drug loss, were obtained by simulation	Inhaler optimization and testing	[123]
	CFD	ANSYS	384 combinations of spray characteristics resulting from the combination of six input spray parameters over a range were investigated.	A time-averaged frozen flow-field method was introduced, thereby reducing the flow-field simulation time and focusing only on the particle trajectory simulations	High-computational-volume simulation	[127]
	CFD	ANSYS	This paper simulated airflow, droplet spray transport and aerosol deposition of pressurized metered dose inhalers in the upper airway	Optimal design of inhalers assessed by lung deposition rates of lung-targeted drugs	Inhaler optimization	[161]
	CFD	Fluent 14.5	The objective of this study was to develop quantitative correlations for predicting the aerosolization behavior of a newly proposed DPI	CFD simulations were conducted to predict both flow field-based and particle-based dispersion parameters that captured different measures of turbulence	Inhaler optimization	[100]
	CFD	ANSYS Fluent	The objective of this study was to characterize the transport and deposition of an aerosol using a combination of CFD and in vitro experiments	Simulations clarified the effect of gas flow regimes (e.g., recirculation flow) on particle transport and deposition	Soft-mist inhalers	[102]
	CFD	OpenFOAM	This paper developed a drug powder shedding model for numerical computation of fluid flow and carrier particle motion in inhalers	The flow field of the inhaler device and the hydrodynamic stresses acting on the carrier particles were simulated and statistically analyzed	Geometry and formulation optimization	[107]
	CFD, DEM	-	This paper numerically investigated the mechanisms of powder de-agglomeration on mechanical impaction	The simulations monitored and quantified information on particle-throat and particle-fluid interactions, debris counts, fine particle fraction, and powder deposition	Powder de-agglomeration	[103]
Nasal-brain-targeted drug delivery	CFD	ICEM-CFD™	A study of the effect of nasal middle-roof surgery on the olfactory delivery of nasal sprays	Computation of drug particle deposition in the olfactory cavity and olfactory bulb, and nasal airflow streamlines	Nasal postoperative drug delivery	[144]
	CFD	OpenFOAM	A workflow was developed to predict personalized human nasal drug delivery efficiency for pulsating bi-directional aerosol delivery	Study of the tilt angle of the drug delivery device clockwise with respect to the horizontal axis to find the optimal air flux	Spray tilt angle	[142]
	CFD	OpenFOAM	Patient-specific nasal geometry was studied and aerosol transport under bidirectional pulsatile flow conditions was explored	Simulations were carried out to calculate the deposition of particles under pulsating and non-pulsating airflow conditions	Pulsatile airflow; drug deposition	[143]
	CFPD	ANSYS	Article examines intra-nasal COVID-19 vaccine droplet transport, deposition and triggering of immune responses in specific regions	Numerical simulations explored the effects of droplet initial velocity and composition as well as spray tracking on vaccine delivery efficiency	COVID-19 vaccine	[166]
	CFPD	ANSYS Fluent	The feasibility of olfactory delivery was tested with two nebulizers (vibrating mesh and jet) and two delivery (normal and bidirectional) techniques	Particle deposition and airflow trajectories in the nasal cavity were simulated	Bidirectional intra-nasal delivery	[167]
	CFD	ANSYS Fluent	In vitro experiments and numerical analyses were used to quantify the effect of nasal dilatation on drug delivery to the nasal airway and olfactory region	Simulations showed the addition of a recirculation zone in the middle passage of the dilated nose, which led to a decrease in pressure and an increase in ventilation in the upper nose	Nasal dilatation; drug deposition	[145]
	CFD	ANSYS	To determine parameter combinations for effective drug delivery of intranasal spray steroids to the ostiomeatal complex and maxillary sinus in patients with chronic rhinosinusitis	Simulation of intranasal airflow and drug particle transport using computational fluid dynamics modeling	Drug delivery in pathologic nasal passages	[146]
	CFD	ANSYS Fluent	Drug particle deposition was studied with auxiliary airflow, spray cone angle, particle size, drug particle delivery velocity and specific injection position as variables	CFD was used to simulate the effect of auxiliary airflow on the deposition of drug particles. DPM was adopted to track the deposition trajectories of drug particles	Optimization of spray patterns	[138]
	CFD	ANSYS	This study elucidated the effects of sinus and reduced turbinate length on nasal flow and particle deposition efficiencies	CFD simulated the transient flow field in the nasal cavity at different flow rates. Particle deposition was investigated using DPM	Study of Nasal Differences	[168]
Whole- lung delivery and deposition of drugs	CFD	OpenFOAM	The most detailed and comprehensive computer simulation of unsteady state transport and deposition of aerosols into the alveolar region	Simulations provided high-resolution 3D spatial and temporal data on aerosol transport and deposition	Aerosol alveolar deposition	[149]
	CFD	ANSYS ICEM 10	The Sar-Gel based method in combination with sectional upper airway casts is a practical approach to visualize regional depositions with nebulizers	Understanding potential mechanisms of particle migration and deposition using numerical simulations	Particle deposition in the upper respiratory tract	[156]
	CFD	ANSYS ICEM	This study presented a methodology to numerically quantify alveolar deposition with continuous particle release by integrating bolus deposition curves.	The simulation computed the cumulative alveolar deposition of particles released continuously over a defined period of time or over the entire inhalation cycle	Alveolar deposition of drugs	[162]
	CFPD	ANSYS	A model that approximates whole-lung geometry and enables physiological boundary conditions was developed to simulate whole-lung deposition of particles	Real respiratory cycles and different inhalation/exhalation scenarios, such as exercise, were simulated, and the results were consistent with experiments	Whole-lung model simplification	[153]
	CFD	Siemens Star CCM + 16.06.010	This work was set out to contribute to the development of a liposomal formulation of erlotinib delivered locally to the lungs.	The aerosol deposition in lungs was calculated by tools of computational fluid and particle mechanics	Drug-containing liposomal delivery	[169]
	CFD, DEM	ANSYS Workbench 2021	The non-spherical particle drug deposition in human airway for drug delivery with the effect of drug size, shape, density, and drug concentration was investigated	The CFD-DEM model simulated the non-spherical drug transport process and achieved a better balance between numerical accuracy and computational volume	model coupling	[170]

## 5. Application of Mechanism Models in the Study of New Drug Dosage

### 5.1. Nano Dosage

There are rare applications of numerical simulation in the research of traditional nano dosage preparation such as the thin-film dispersion method and the ethanol injection method. In recent years, with the application of microfluidics in nano dosage preparation, numerical simulation plays an important role in microfluidics preparation. Microfluidics provides a new strategy for the preparation of nano dosage. Microfluidics allows precise manipulation of tiny fluids with volumes in the range of nanoliters to arils through microtubules with diameters in the range of tens to hundreds of micrometers. Traditional nano dosage preparation suffers from slow mass transfer, poor homogenization, wide particle size distribution, and poor preparation reproducibility [171,172]. Compared with the traditional nano dosage preparation, the microfluidic preparation exhibits significant advantages. The micron-sized channels of microfluidics are characterized by small volume, large ratio surface, fast mass and heat transfer, low reagent consumption, and precise control of the physicochemical properties of nanoparticles [173,174]. In experiments, researchers can obtain very limited information of fluid behavior using microfluidics [175]. Numerical simulations can comprehensively reveal key information such as mixing mass transfer, pressure distribution, fluid flow pattern and velocity distribution. Numerical simulations become an efficient tool to simulate experiment processes and results. Microfluidics use CFD methodology. Because the fluid presents laminar state in the tiny channel of microfluidics, the physical field of laminar is mostly used in microfluidic simulation. The commonly used software includes ANSYS Fluent and COMSOL Multiphysics. By searching relevant literature on Web of Science and PubMed, we found that numerical simulations are most applied to the preparation of liposomes and nanoparticles. Subsequently, we will review the simulations of these two dosage forms, respectively. Table 4 summarizes literatures related to numerical simulation mechanism modeling of nano dosage at the end of this section.

#### 5.1.1. Liposomes

**Figure 11:**
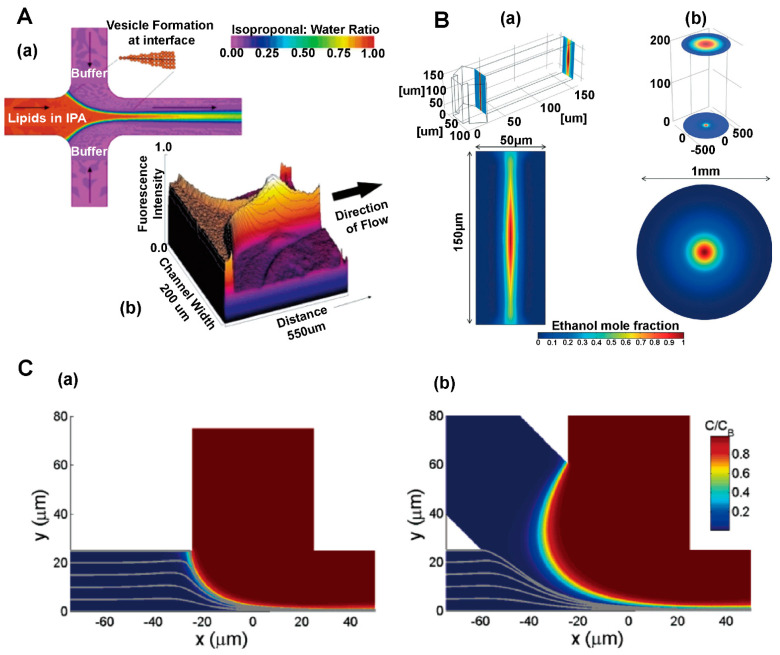
**(A**) (**a**) Schematic of liposome formation process in the microfluidic channel. Color contours represent the concentration ratios of isopropyl alcohol (IPA) to aqueous buffer. (**b**) 3D color contour map of fluorescent dye DiIC_18_ fluorescence intensity at focused region during liposome formation. A ridge of increased fluorescence is clearly visible along the boundary between the IPA and buffer [176]. (**B**) CFD simulation of ethanol concentration in (**a**) 2D-MHF vs. (**b**) 3D-MHF [178]. (**C**) Two-dimensional simulation results of two different mixer designs. (**a**) One-half of the mixer with two orthogonally crossed channels. (**b**) One-half of the mixer with diagonal channels [179].

**Figure 12:**
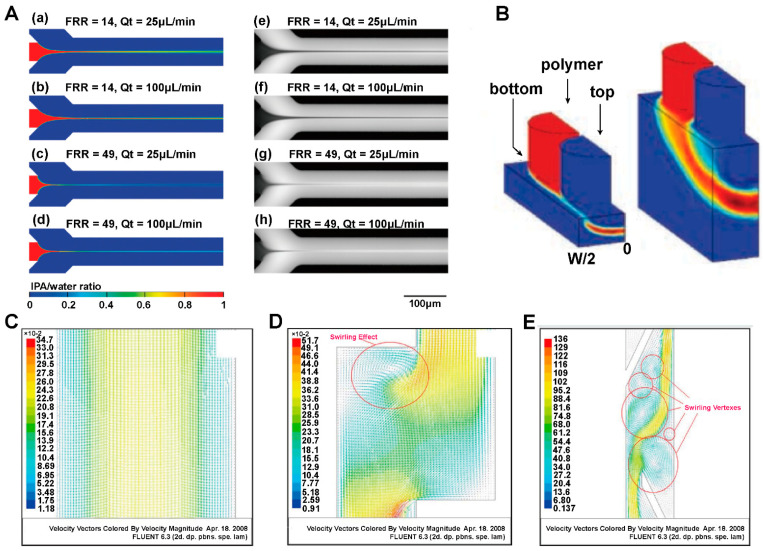
(**A**) Numerical simulation (**a**–**d**) and experimental (**e**–**h**) flow patterns of liposomes prepared by microfluidic focused flow under different FRR and total flow rate (Q_t_) conditions [177]. (**B**) 3D simulations with different channel aspect ratios (w/h), 3.33 (left) and 0.83 (right), respectively. There are three inlets include bottom, polymer and top [180]. (**C**–**E**) Velocity vector plots for (**C**) Geometry 1, (**D**) Geometry 2, and (**E**) Geometry 3 [181].

In an early study of microfluidic preparation of liposomes, Jahn et al. [176] used CFD to map the mixing concentration gradient diagram of isopropanol and water during microfluidic liposome preparation as shown in Figure 11A. The diagram revealed the fluid mixing state of the oil and water by using fluid-focused preparation method. In another paper, Jahn et al. [177] compared the simulation and experiment results of microfluidic fluid morphology, as shown in Figure 12A. They found that the two morphologies are in high agreement, thus verifying the accuracy of the numerical simulation. Subsequently, Jahn simulated the flow profile when the side channel was set as 90° and 45° to the central channel. Then, they considered two-phase flow rate ratio (FRR) and channel width as variables, and found the decreasing trend of the ethanol concentration gradient with the increase in mixing time. The decreasing trend explain the precipitation and self-assembly processes when lipid solubility decreases to a critical value.

Hood et al. [178] also used CFD to elucidate the focusing morphology of the fluid. They developed a novel microscale device that utilizes three-dimensional microhydrodynamic focusing (3D-MHF) to realize one-step continuous flow assembly of monodisperse nanoscale liposomes in concentric capillary arrays. As shown in Figure 11B, they compared the difference between two-dimensional microhydrodynamic focusing (2D-MHF) and 3D-MHF. The velocity contour plots showed that in the 2D rectangular channel, the fluid velocity was not uniformly distributed in the vertical plane, especially near the edges of the rectangle, namely the “edge effect”. Because of the edge effect, there was a significant difference of fluid velocity between the edge and the center of the channel. In contrast, the fluid focusing in the 3D channel showed a more uniform velocity distribution. Park et al. [179] investigated the effect of the microfluidic channel on the fluid mixing. They added an additional fluid channel at the angle of 45° between side channels and central channel to interfere the fluid mixing. The contour plots of the results showed that without interfering channel, macromolecules flowing along the outer flow line contacted with the reactants earlier than molecules flowing along the inner flow line, leading to drastic changes in concentration around diffusion. However, with interference channels, the fluid mixed well, as shown in Figure 11C.

#### 5.1.2. Nanoparticles

The application of numerical simulation mostly focuses on the studies of mixing the characteristics of nano precipitation method in microfluidics. By using CFD, researchers enable to obtain key information such as fluid mixing contour plots, pressure contour plots, and velocity vector diagrams. The above parameters reveal the effects of preparation conditions on the fluid mixing efficiency. The mixing efficiency affects the size of the nanoparticles. Naher et al. [181] analyzed the fluid mixing efficiency in microfluidic nanoparticle preparation by using CFD in 2010. They thoroughly investigated the fluid properties and mixing efficiency under three different geometric microfluidic channels. Naher also used velocity vector plots to demonstrate the flow paths of the fluids and evaluated the mixing efficiency by calculating the mass fraction of the fluids, as shown in Figure 12C–E. The results showed that the geometry of the channel had a great impact on the mixing behavior with the constant inlet velocity, fluid pressure and fluid properties. Rhee et al. [180] used COMSOL Multiphysics to simulate the fluid dynamics of 3D focusing, and optimize the design of microfluidic channels for the preparation of nanoparticles. They focused on the size and location of the inlet, as well as the aspect ratio of the channel (i.e., the ratio of width to height w/h). These variables determined the fluid morphology of 3D focusing in the channel and had an impact on the preparation of nanoparticle. Taking the width-to-height ratio as an example, when w/h < 2, the channel exhibited a flat concentration distribution. When w/h > 2, a “banana”-shaped distribution occurred, with a long tail that extends to the top wall, as shown in Figure 12B. The “banana”-shaped distribution was more prone to cause aggregation of nanoparticles and lead to the failure of nanoparticle suspensions. Rahimi et al. [182] investigated the preparation of curcumin nanoparticle under different channel angles. They constructed three channel intersection angles of 45°, 90°, and 135° and confirmed that the nanoparticles had the smallest size and lowest dispersion at 135° angle in experiments. Meanwhile, the numerical simulation also showed that the fluid mixing was more uniform at 135° intersection angle. This phenomenon suggested that the size of the nanoparticles may be highly related to the uniformity of mixing. In 2017, Cheng et al. [183] constructed a model with triple coupling of multiphase CFD, population balance equation (PBE), and micro-mixing modeling. The triple coupling model can simulate the effect of the presence of solid crystals on the crystalline properties in the nanoparticle preparation. They successfully integrated the general discretized PBE and multi-environmental micro-mixing model into a multiphase flow mixing model and developed a multiphase solver based on OpenFOAM. With this solver, they investigated important parameters such as mixing fractions, volume fractions, concentration distributions and crystal content distributions of substances under different variables. In addition, related studies are systematically summarized and organized in tabular form for convenient reference.

**Table 4:** Summary of the literature related to numerical simulation mechanism modeling of nano dosage.

	Mechanistic Model	Simulation Platform	Content of Research	The Role of Simulation	Research Focus	Reference
Review	-	-	An overview of nanoparticle preparation methods, particle types, model simulations	Simulating fluid mixing to induce nanoprecipitation	Nanoparticle Preparation	[175]
	-	-	Modelling and simulation of nanoparticle pharmaceutical preparation process technology	A long-term vision for integrating drug delivery modelling with predictive models for product and process design was presented	Summary and outlook of the model	[184]
	-	-	This review focused on transport phenomena and hydrodynamics involved in mixing for enhanced mass transfer during antisolvent precipitation of synthetic nanomedicines in microfluidic systems		Mixed process research	[185]
Liposome	CFD	-	Controlled vesicle self-assembly in microfluidic channels using hydrodynamic focusing technology	Use contour to clarify liposome formation process in the microfluidic channel	Self-assembly of liposomes	[176]
	-	COMSOL Multiphysics 3.4	Investigating the formation of liposomes up to tens of nanometers in diameter by microfluidic methods	Simulations explored the use of intermittent solvent injection in microfluidic preparation of liposomes	Preparation conditions study	[177]
	-	COMSOL Multiphysics 3.5a	Freezing self-assembled continuous flow in microfluidic devices for imaging liposome formation	Simulation to obtain qualitative concentration profiles, flow rate profiles, ellipsoidal concentration profiles for liposome preparation	Liposome preparation method	[186]
	-	-	Microfluidic synthesis of PEG and folate conjugated liposomes for one-step formation of targeted stealth nanocarriers	Numerical simulations showed a gradual decrease in the mole fraction of ethanol flowing along the center of the channel	Liposome preparation process	[187]
	CFD	COMSOL Multiphysics 4.2	A facile route to the synthesis of monodisperse nanoscale liposomes using three-dimensional microfluidic hydrodynamic focusing technology	Simulated contour plots exploring the velocity distribution in the cross-section of 2D and 3D focused flows	3D microfluidic fluid focus	[178]
	-	COMSOL Multiphysics 4.2	High-throughput continuous flow production of nanoscale liposomes based on microfluidic focusing technology	Modelling changes in lipid-containing phase concentration in microfluidic channels with different aspect ratios	High-throughput microfluidics	[188]
	-	COMSOL Multiphysics	Hydrodynamic focusing prior to uniform mixing of two phases in a microfluidic device	Simulated contour plots showed contact mixing between different fluids in a microfluidic channel	Microfluidic fluid mixing	[179]
	-	COMSOL Multiphysics	Synthesis of size-tunable polymer nanoparticles by 3D hydrodynamic flow focusing in microchannels	Simulated fluid flow regimes in microfluidic channels with different aspect ratios, which correlate with nanosuspension stability	Optimizing process parameters	[180]
	-	COMSOL Multiphysics 5.5.	Precise control of liposome size using characteristic time depended on solvent type and membrane properties	Simulation calculation of ethanol concentration decrease curve with time for different focusing flow rate ratios of microfluidics	Factors affecting liposome size	[189]
	-	-	Recorded two-dimensional top view of tiny droplet formation in a T-shaped microfluidic device with necking	Velocity vector plots illustrated the change in velocity and direction during t-channel droplet generation	Droplet microfluidics	[190]
	-	COMSOL Multiphysics	Continuous flow production of injectable liposomes by microfluidic methods	Simulated mixing of oil and water phases at different microfluidic channel crossing angles and total flow rates	Microfluidic two-phase mixing	[191]
Nanoparticle	CFD	Fluent	Effect of micro-channel geometry on fluid flow and mixing	Simulations were performed to obtain the flow path and mixing efficiency of the fluid in microfluidics.	Microfluidic fluid mixing	[181]
	CFD	ANSYS Fluent	Mixing properties of poorly water-soluble drugs studied by microfluidic-assisted nanoprecipitation	Smaller dispersion and homogeneous mixing at 135° microfluidic channel angle	Factors affecting nanoparticle size	[182]
	CFD	ANSYS Fluent	Simultaneous experimental and CFD simulation studies were performed to prepare uniform, reproducible and stable PLGA nanoparticles	Observation of fluid flow characteristics and micro-mixing in microfluidic systems by CFD simulation	Advantages of the microfluidic approach	[192]
	CFD	Ansys CFX 18.0	Elucidation of the fundamentals of drug encapsulation in polymeric NPs by microfluidic nanoprecipitation methods	The simulations investigated the effects of solution/water flow velocity ratio and microchannel geometry on the nanoprecipitation process.	Synthesis of drug-carrying nanoparticles	[193]
	Residence time distribution based PBM	-	A model for the liquid-phase synthesis of nanoparticles in two common continuous milli reactors was presented	Simulations revealed that residence time distribution effects are important for predicting the standard deviation of particle size distributions	Factors affecting nanoparticle synthesis	[194]
	CFD-PBM	ANSYS Fluent 15	Towards the nucleation rate prediction of curcumin particles in microfluidic assisted nanoprecipitation	In the simulation of the nucleation process, particles with larger sizes were mainly distributed along the reactor axis	Predicting nanoparticle size, dispersion	[195]
	CFD-PBE	Fluent	Simulation of antisolvent crystallization in impinging jets with coupled multiphase flow-micromixing-PBE	Coupling CFD and PBE to aid the experimental design of mixing-sensitive crystallization processes	Nanoparticle crystallization process	[183]

### 5.2. Microneedle

The mechanistic model has a wide application in the study of microneedle drug delivery processes. Numerical simulation effectively reduces the time and experiment costs. Researchers can use numerical simulation to evaluate the design of microneedles (MNs) in terms of geometry and material parameters, and test and optimize the existing MNs [196]. Numerical simulations of MNs can be summarized as follows: evaluation of the material properties of MNs [197,198,199,200]; breakage assessment of the structural morphology of MNs [201,202,203]; exploration and modeling of the mechanical properties of the skin [204,205,206]; evaluation of the effect of drug release after puncture [207,208,209]; and assessment of drug transport within the skin [208,210].

The numerical simulation methods for MNs can be divided into three categories based on the previous research. CFD models are commonly used to simulate the fluid flow, when studying the drug flow within MNs and drug flow from MNs to skin. The FEM, FDM and FVM are commonly used to calculate the distribution of drug concentration in each region of the skin, when studying drug diffusion within the skin. The constitutive model is used to simulate stress and strain, when calculating the mechanical behavior of skin compression during puncture. There are two kinds of tools for MN simulation include in-house programming and commercial software. (i) An in-house java programming tool was used to test the optimization of the squared MN patch [211]. MATLAB was used to acquire skin pore profiles from histological images and simulate trajectories and penetration depth of micro-particles delivery by gene gun into MN-pierced skin [212,213]. (ii) Commercial software such as COMSOL [200], ANSYS [214,215], Preview [216] and ABAQUS [217] was used to simulate the diffusion profiles in the computational domain. Table 5 summarizes literatures related to numerical simulation mechanism modeling of microneedles at the end of this section.

#### 5.2.1. Exploration of Microneedle Property Parameters

The properties of MNs cover a variety of aspects such as needle materials, needle structural morphology, and microneedle array size. Buckling is the main mode of MN failure at microneedles puncture procedure [218]. Elastic parameters such as Young’s modulus and Poisson’s ratio play an important role in predicting the geometry deformations of MNs. The mechanical properties of materials are determined by a combination of parameters. For example, Juster et al. [219] noted that the mechanical strength, elastic modulus, and fracture toughness reflect the insertion ability of the polymer-based MNs. Yan et al. [220] conducted a simulation of the mechanical properties of a 6 × 6 microneedle array at skin puncture procedure. The structural mechanics module of COMSOL Multiphysics was used to define stress analysis. They used static analysis to simulate von Mises stress and microneedle/skin deformation under static equilibrium. It was found that the values of compression breaking force and puncture force did not adequately characterize the mechanical properties of MNs. The characterization of mechanical properties of MNs that made in different materials correlates more strongly with von Mises stress.

**Figure 13:**
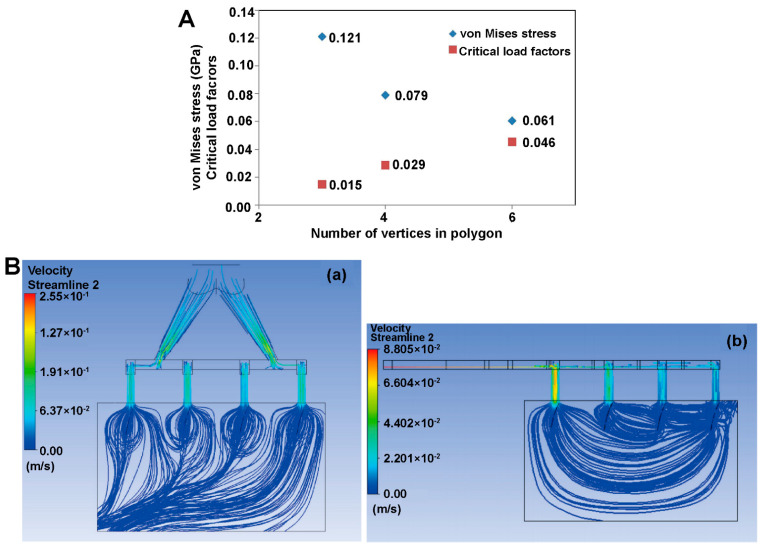
**(A**) Von Mises stress and critical load factors for 3 × 3 arrays of MNs with triangle, square and hexagon base geometries [225]. (**B**) Velocity flow diagrams of drug delivery inside MNs of two geometries. (**a**) Vertical flow delivery. (**b**) Lateral flow delivery [207].

Considering the efficiency of modeling and the low cost of trial and error, numerical simulation has become an effective tool to study the geometry of MNs. Numerous researchers have used the FEM to investigate the effects of different microneedle sizes [221], tip shapes [222,223], and material properties [202,224] on their mechanical properties. For example, Loizidou et al. [225] investigated the effect of microneedle arrays with different polygonal foundation on the puncture ability of MNs. As shown in Figure 13A, the simulation indicated an inverse relationship between the number of vertexes in the polygonal foundation and the von Mises stress value. The results meant that as the number of vertexes in the polygonal structure increased, the value of von Mises stress on individual microneedles decreased. The relationship indicated that for the same number of microneedles, the more vertexes at the foundation of the microneedle array, the higher compressive load the array could withstand.

#### 5.2.2. Construction of the Skin Model

Skin models are constructed to test the skin puncture to test the puncture ability and the drug delivery ability [203,226]. A number of skin mechanics tests have been performed to obtain the parameters of the basic physiological properties of the skin for modeling. Various mechanical properties of skin are studied such as anisotropy, Young’s modulus, stiffness, compressibility, strength, toughness, initial stress, and skin friction coefficient [227]. It is important to consider mechanical properties of skin such as different species or the same specie with different gender, age, race, and body parts, when applying them to modeling [228]. In addition, the skin is composed of epidermis, dermis, and subcutaneous tissue. We should consider the different mechanical properties of the different skin layers and the interactions between the layers while modeling [229]. By skin modeling and simulation, it is possible to know the maximum stresses on the skin and the maximum puncture depth, which are necessary for MN design.

The constitutive model is a commonly used for skin modeling. In the simulation, physiological values are usually associated with the parameters of the constitutive model, which are used to define the compressive deformation behavior of the skin in the simulation. The simulation results of the constitutive model agree well with the experiment results [206], and the detailed model setup can be learned in the review by Yan et al. [230]. Meanwhile, some researchers consider the skin as an aqueous medium, which is used to calculate intradermal drug diffusion rate and local pressure. Henriquez et al. [207] modeled the skin as an aqueous medium and simulated the flow of analgesics within the skin after puncture by using CFD. Figure 13B showed the structural design of two MNs. Figure 13B(a) showed the vertical flow delivery of drug from the reservoir to the MN tip, and Figure 13B(b) showed the lateral flow. When the drug is delivered vertically, the velocity line diagrams showed the greater depth of intradermal delivery, and the distribution towards the capillary flow is better. The construction of skin model can realize the prediction of skin puncture and drug release performance at low cost.

#### 5.2.3. Simulation of Microneedle Drug Release

**Figure 14:**
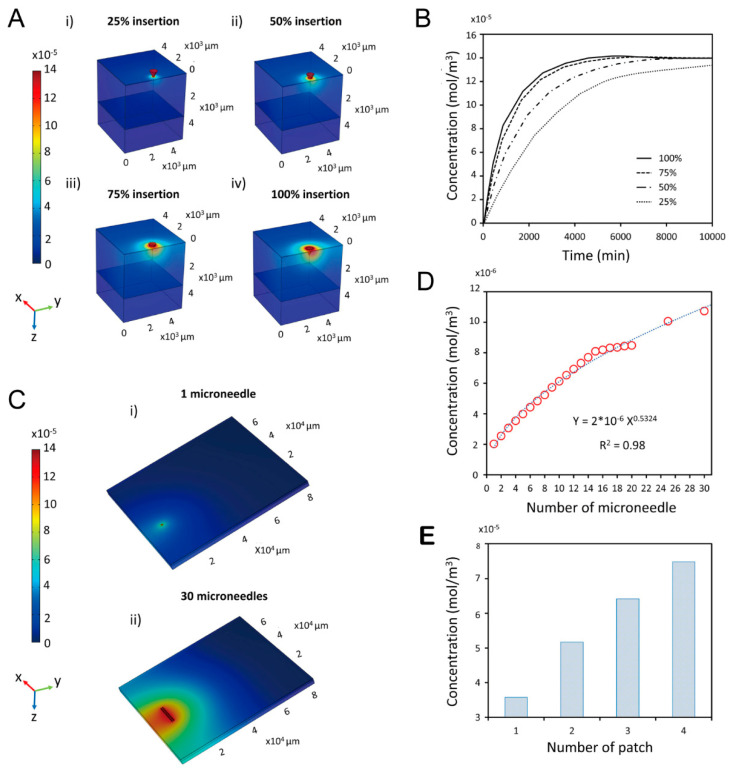
COMSOL simulation demonstrated that the transdermal drug delivery increases in function to the increment of the number of microneedles on the surface patch and percentage of penetration per microneedle. Effect of penetration % of one microneedle into the skin. (**A**) Insertion of one microneedle into the skin. (**i**) 25% insertion, (**ii**) 50% insertion, (**iii**) 75% insertion, and (**iv**) 100% insertion. (**B**) Transdermal drug delivery versus % of penetration of one microneedle into the skin. Effect of the number of microneedles. (**C**) Concentration profile of microneedles into the skin. (**i**) one microneedle, (**ii**) thirty microneedles. (**D**) The tendency of transdermal drug delivery versus the number of microneedles into the skin, empirical eq. (**E**) Transdermal drug delivery versus the number of microneedle patches penetrating the skin [226].

Researchers need to monitor the safety, solubility and biocompatibility of MNs, as well as the drug release rate after puncture. These properties of MNs are essential in the clinic [231]. Compared with traditional biomechanical methods, numerical simulation has the advantages of fast simulation speed, easy manipulation, low economic cost and accurate results. Due to the advantages, simulation is a good tool to predict MN matrix degradation and drug release [232]. Zoudani et al. [203] simulated the dissolution process of dissolving microneedles in porous media. They assessed the effect of initial drug loading and spacing size of microneedle arrays on MN dissolution and drug release. After optimization of the microneedle arrays, complete drug release needed shorter time than before. The residual concentration increased in the tissue. Benslimane et al. used FDM to derive a numerical solution for the distribution of the drug concentration in the skin after drug release. Then, they investigated the effect of the diffusion coefficient, initial concentration and device length on the concentration diffusion. The results revealed the influence of the different parameters studied on diffusion and concentration over time and space. With different material microneedle arrays, David et al. [226] evaluated their chemical composition, uniformity of physical characteristics, capacity to penetrate the skin, and response to thermal and thermo-mechanical changes. Contour plots in Figure 14 showed the distribution of drug concentration with different depths of penetration and different numbers of punctures. They plotted the relationship of drug concentration with the number of microneedles, the number of groups and time based on the data obtained from the simulation. The simulations evaluated the dissolution and drug release process of MNs after penetration, and give valuable guidance for the geometry design of microneedle, the selection of materials and the quantity of initial drug loading.

#### 5.2.4. Modelling of Intradermal Drug Transport

Several models are used to simulate intradermal drug transport, which can be classified into the FDM, FEM, and FVM based on the differences in spatial discretization. Within a small margin of error, the three mechanistic models described above will give the same results. FDM has the advantage of maximizing accuracy and minimizing instability. The FEM enables the simulation of complex geometries, disordered structures and non-uniform media by controlling the mesh number in different regions of the geometrical model. FVM is ideally suited for discontinuity models. Hybrid models are common discontinuity model. Regarding the various application scenarios of the FEM, Mitragotri et al. [205] gave a case of skin penetration by using the FEM. Khanday et al. [233] considered the absorption rate of drug components as a decreasing function from the drug release point to the target site. They used MATLAB and the FEM to calculate and obtain the drug concentration at different dermal grid nodes, as well as plotted the drug concentration versus dermal thickness. Römgens et al. [234] used a combination of the FEM and photobleaching experiments to probe the diffusion coefficients of macromolecules in the epidermis, papillary dermis, and reticular dermis to evaluate the transport of macromolecules through the various skin layers.

**Table 5:** Summary of the literature related to numerical simulation mechanism modeling of microneedles.

	Mechanistic Model	Simulation Platform	Content of Research	The Role of Simulation	Research Focus	Reference
Review	FEM, FDM	COMSOL	This article introduced FEM research for microneedle transdermal drug delivery systems, focusing on microneedle design strategy, skin mechanics models, skin permeability, and the FEM research on drug delivery by MNs		The whole process of drug delivery	[230]
	-	CAD	This review assessed the various materials involved in the fabrication of MNs as well as incorporation of other excipients to improve drug delivery for novel medical devices		Microneedle preparation materials	[197]
	-	CAD	This review discussed different microneedles such as solid, hollow, dissolved and coated microneedles categorized on the basis of the type of transport, as well as preparation techniques for microneedles		MN manufacturing technology	[199]
	FDM, MOL, FEM, FVM	-	The article reviewed the application of numerical methods to diffusion-based modeling of skin permeation mechanisms. Key findings and insights of reviewed models were highlighted		Skin permeation model	[204]
	FEM	-	The review explicitly showed the various attempts to model drug transport within the viable skin. Furthermore, a brief review was conducted on the different models that explained stratum corneum transport, microneedle dynamics and estimation of the diffusion coefficient		Intradermal drug transport	[210]
	FEM	-	This article illustrated trends and advances in mathematical modeling, as well as simulation and optimization of microneedles for transdermal drug delivery		Advances in MN numerical simulation	[196]
	FEM	-	An overview of recent developments for microneedles made using injection molding and hot embossing techniques for active transdermal drug delivery. The paper covered microneedle array design and microneedle application simulation		MN manufacturing technology	[219]
	FDM, FEM	-	This paper provided an overview of various approaches to modeling mathematical models of skin permeability, as well as their advantages, limitations, and future prospects		Skin penetration model	[205]
Microneedle property	FEM	ANSYS	This study fabricated and evaluated in vitro 3D printed microneedles for transdermal drug delivery	The article used CAD to design microneedle size and shape, and used FEM to simulate the MN insertion process and penetration process	3D printed microneedles	[201]
	-	COMSOL	The formulation and evaluation of a chitosan microneedle patch for the transdermal delivery of meloxicam to manage pain in cattle	Simulation of transdermal drug delivery process by microneedle patch system	Chitosan microneedle patch	[226]
	FEM	COMSOL	The article discussed the application of microneedles coated with thin films of drug solutions and determined the effect of microneedle geometry on drug permeability	Effective epidermal permeability was calculated for a range of microneedle shapes and sizes, and the optimal geometry was determined	Geometry acts on skin permeability	[212]
	FEM	Auto FEM Lite	This study used biopolymers extracted from fish scales to prepare microneedles and confirmed MNs’ ability to penetrate the skin	The authors constructed a 3D biopolymer film microneedle geometry model and calculated its mechanical properties	New material	[200]
	FEM	ANSYS	The effect of shape of the microneedle on the flow inside the micropump	Modeling and numerical analysis of microneedles with different shapes	MN shape acting on the fluid	[214]
	FEM	FEBio	This paper quantified the mechanical properties of skin in vitro and in vivo to optimize microneedle design	The study developed a finite element model of the skin, simulated in vivo skin indentations, and modeled the deflection of the skin during applied pressure loads	Geometry optimization	[216]
	FEM	ABAQUS	A computational model to predict the optimal design of the microneedles and their arrangement within an array	Microneedle and skin dynamics were modeled. The simulations revealed the importance of geometric parameters in inducing immune responses	Geometric shapes in immune response	[217]
	FEM	COMSOL	Mechanical properties of sugar microneedles and their ability to deliver drugs to the skin	Simulation of individual microneedles and finite element analysis of surface von Mises stresses	Sugar microneedles	[218]
	FEM	-	A new computational approach for modeling and evaluation of novel dissolving microneedles for drug delivery	Numerical simulation of the dissolution process of dissolving microneedles in porous media	Dissolving microneedles	[203]
	FEM	ANSYS	the fabrication method of hollow out-of-layer hafnium oxide (HfO2) microneedles mainly based on deep reactive ion etching of silicon and atomic layer deposition of HfO2	Hollow microneedle finite element modeling, skin hollow microneedle array finite element modeling	Materials exploration	[202]
	FEM, CFD	ANSYS	This paper presented a new design of hollow, out-of-plane polymeric microneedle with cylindrical side-open holes for transdermal drug delivery applications	Axial and lateral loads and fluid flow during microneedle skin insertion were investigated using the FEM and CFD, respectively	Geometry optimization	[215]
Skin mode	FEM	FEBio	The aim of this study was to develop a predictive anisotropic, hyperelastic constitutive model of human skin and to validate this model using laboratory data	The model described the anisotropy of mechanical properties of living mouse and human skin during uniaxial stretching along three load axes	Skin anisotropic hyperelasticity modeling	[227]
	FEM	ABAQUS	A numerical simulation of the insertion process of the microneedle into human skin was reported using the finite element method	A multilayer finite element model of the skin was developed to simulatively probe microneedle-skin interactions	Mechanical properties of skin	[223]
Microneedle drug release performance	-	MATLAB	This paper investigated the process of simulated microneedle drug delivery nanoparticle-assisted particle delivery, in particular the penetration depth of the particles	Models were used to simulate the velocity and trajectory of particles as they penetrate the target	Model of particle transport by MNs	[213]
	FEM	COMSOL Multiphysics	Finite element analysis for biodegradable dissolving microneedle materials on skin puncture and mechanical performance evaluation	The article simulated the entire process of a single microneedle penetrating the skin	Material-penetration	[220]
	FEM	COMSOL Multiphysics	Evaluation of geometrical effects of microneedles on skin penetration by CT scan and finite element analysis	The flexibility of individual microneedles and the penetration force of microneedle arrays were simulated	Penetration performance	[225]
	FEM, CFD	ANSYS	Two analgesic drug delivery schemes focused on hollow microneedle arrays were evaluated by CFD simulations	Modeling drug flow and microneedle drug delivery to simulate the potential for drug entry into porous aqueous media	Drug delivery applications	[207]
	FDM, Runge-Kutta methods	MATLAB	Drug-loaded degradable conic microneedles were modeled to characterize the degradation rate and drug release profile	Intradermal degradation rates and drug release profiles of microneedles obtained by simulations	Microneedle degradation and drug release	[232]
Intradermal drug delivery	FDM	MATLAB	This work used a one-dimensional non-stationary diffusion model to calculate the drug concentration field after microneedle transdermal drug delivery	The effect of the diffusion coefficient, initial concentration and device length on the concentration diffusion was investigated by simulation	Model exploration	[208]
	FEM	MATLAB	Study on drug absorption rate in different compartments of transdermal drug delivery system by variational finite element method	The FEM studied the diffusion process of transdermal drug delivery and calculated the drug concentration in the dermis	Dermal drug diffusion	[233]
	FEM	ABAQUS	This study assessed the transport of macromolecules through and within various human skin layers	Skin modeling to simulate intradermal drug diffusion	Macromolecular intradermal transport	[234]

### 5.3. Gelling Agent

Since 2019, researchers have applied numerical simulation to periodontal drug delivery. They investigated gel sustained-release dosage form for slow-release delivery and long-lasting antimicrobials in periodontal pockets. The cases of periodontal pocket gel drug delivery applying numerical simulation can be divided into three categories, including studies of periodontal pocket gel filling, studies of dissolution chamber modelling and studies of the release process of drug molecules. Among the three categories, the former two categories use macroscopic simulation models, such as CFD, and the last category uses molecular dynamics (MD). Numerical simulation of gels is still in its early stages with fewer research, and need more researchers to devote themselves to it.

**Figure 15:**
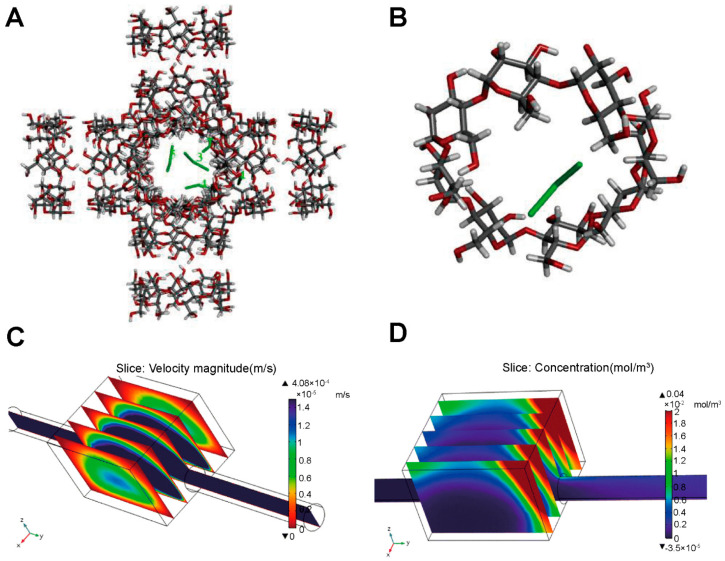
**(A**) The molecular docking conformation of I^3−^ @cross-linked cyclodextrin metal-organic framework. (**B**) The molecular docking conformation of I^3−^ @cyclodextrin. (**C**) Steady-state flow velocity. (**D**) Drug concentration profiles at 60 min in the 0.06 mL dissolution chamber with 10 μL/min flow rate and diffusion coefficient of 5 × 10^−6^ cm^2^/s.

Levrini et al. demonstrated the capacity of a new periodontal gel to occupy the spaces inside the periodontal pockets through CFD. Levrini simulated the filling process with gel. During the simulation, they applied two kinds of probes with different outlets. They found the rheological properties of the gel were related to the shape of the periodontal pocket and the kind of the probe [235]. A few researchers have studied the release behavior of gel formulations in periodontal pockets. Senarat et al. explored mathematical modeling of levofloxacin HCl release from zein-based in situ gel using the cup method, aiming to simulate the drug release behavior of in situ gel. They gave scripts for the forward difference formula used in the study and scripts for the central difference formula used to approximate derivatives. Simulation results are in good agreement with experimental drug release data [236]. In addition, Lu et al. [237] designed effective therapeutic system to periodontitis using cross-linked cyclodextrin metal-organic framework (COF) as carrier for iodine and further suspended in hydroxyethyl cellulose gel as I_2_@COF-HEC hydrogel. Lu identified the interaction of iodine with both COF central cavity and individual cyclodextrin moieties of COF by using molecular modelling, as shown in Figure 15A,B. Meanwhile, in vivo pharmacological evaluation further revealed that I_2_@COF-HEC hydrogel can effectively reduce alveolar bone resorption, DPP and periodontal inflammation. The results showed that COF enables a slow and sustained release of iodine in artificial saliva in the oral environment. Furthermore, there are some researches focus on establishing a small dissolution chamber model in the size of a periodontal pocket. The dissolution chamber model can help pharmaceutical scientists design dissolution experiments and determine the dissolution behavior of drug delivery systems in periodontal pockets. Ren et al. [238] developed a dissolution model for long-acting periodontal drug products to mimic the small volume and slow flow rate in the periodontal pocket, as shown in Figure 15C,D. Computer simulations and experimental results showed that drug clearance from the dissolution chamber was fast compared to drug release from the periodontal product. Wanasathop et al. [239] modified and evaluated the dissolution model in the present study for its ability to provide biorelevant and dose-discriminating testing with three drug products, Arestin, Atridox, and PerioChip, of three different dosage forms, microsphere, in situ forming gel, and gelatin chip, respectively. They determined the drug recovery, stability and drug release profiles of the three products by experiment and simulated the change in drug release rate upon blocking the large release surface.

## 6. Applications of Molecular Dynamics in Pharmaceutics

Particle-based simulation methods are currently being applied to drug design [240], design of biocompatible frameworks [241], engineering structures with the ability of cargo carriage [242,243], crystallization and purification of pharmaceutical compounds or low-density structure with the potential applications in drug delivery and the drug release [244]. Regarding the general interest in particle-level simulation, this paper focuses on the pharmaceutical-related and widely used molecular dynamics simulation.

In pharmaceutical field, MD are widely used for drug dissolution, crystallization, absorption, translocation and the arrangement and binding of drug molecules. MD is a mechanism model, which performs force analysis at the molecular and atomic level to explore the details of molecule dynamics. Based on Newton’s laws of motion, MD studies the motion of molecules and intermolecular interactions at the microscopic level [240]. MD simulations calculate the microscopic force and the total energy of the system to obtain the position, velocity and trajectory of a particle over time. Various physical properties of molecules or atoms can be obtained through simulations, including the calculation of free energy and kinetic. The results can be used to calculate the solvation and hydration free energies and chemical potentials [245]. The solvation, hydration free energy and chemical potential are associated with processes such as drug dissolution, crystallization, intermolecular aggregation and depolymerization.

A widely used software package in the field of molecular simulation is Amber Tools [246]. Amber Tools is a free and comprehensive software package to study the structure and dynamics of biological macromolecules such as proteins and nucleic acids. Amber Tools includes several commonly used packages, such as Sander (pmemd), Leap, AnteChember, Parmachk2, and Cpptraj. Sander (pmemd) is the core program for MD simulations. Sander can be used to handle various types of simulations, including standard molecular dynamics, free energy perturbations, and thermodynamic integrals. Leap is used to build simulation environments, such as building solvent environments and adding ions and electric charges to the environment. AnteChember is used to create topologies and parameters for small molecules or modified amino acid residues. Parmachk2 is used to generate small molecule parameters in GAFF format. Cpptraj has trajectory analysis capabilities. There are a number of other commonly used software in the field of MD, including Chemistry at HARvard Molecular Mechanics (CHARMM) [247,248], NAMD [249,250], GRO-ningen Machine for Chemical Simulations (Gromacs) [251], ACEMD [252], and Desmond [253].

This paper focuses on the application of mechanistic modelling to drug formulation. Drug absorption and transport will not be covered. This chapter focuses on the application of MD in drug dissolution, crystallization, oral drug delivery, liquid, suspension and semisolid dosage form.

### 6.1. Application of Molecular Dynamics to Drug Physicochemical Properties

**Figure 16:**
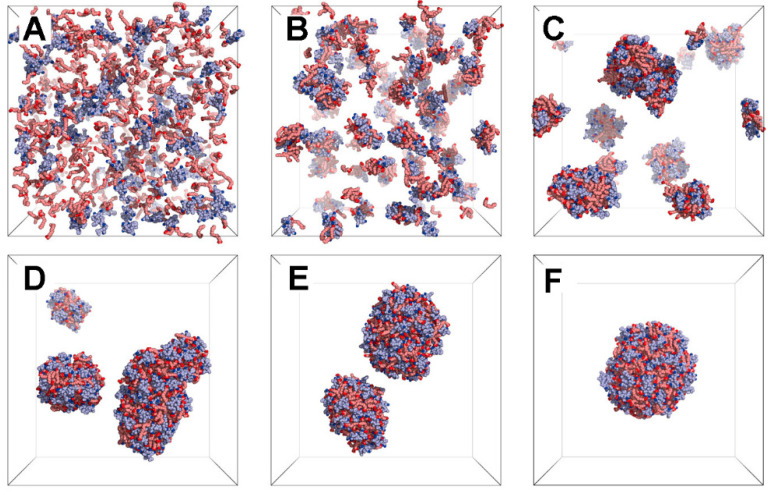
Kinetic visualization of single, stable cluster forming of 100 polymyxin B molecules. Visualization of the large system (500 oleic acid/100 polymyxin B) in water/methanol solvent at (**A**–**F**) 0, 10, 100, 500, 1000, and 1700 ns.

It is essential that the API of the drug has a high solubility. Due to the importance of API solubility, researchers have worked on developing solubility prediction methods to increase the efficiency of drug design. Molecular simulation has been applied to predict the drug dissolution and crystallization properties. Researchers can predict the properties of compounds, as well as understand the factors affecting solubility at the molecular or atomic level [254,255]. According to the classification by Hossain et al. [245], the methods that have been successfully used to calculate drug solubility including Free energy calculations, the Widom insertion method, solubility calculations in the grand canonical ensemble, free energy methods for calculation of molecule partitioning, the Flory-Huggins theory and solubility parameters and equations of state parameters from molecular simulation. These methods have been widely used for the calculation of solvation and hydration free energies in different systems. Kozuch et al. [242] used molecular simulations and experimental methods to study a model system of polymyxin B and oleic acid. Kozuch explored the effects of solvent composition, particle size and charge ratio on the drug molecule hydrophobicity, morphology and stability by modelling small hydrophobic ion pairings, as shown in Figure 16. The simulation results are in good agreement with the experimental results. For drug crystallization, MD explores the reason behind the lack of crystallinity at the molecular and inter-molecular level. Simulations can reveal nucleation obstacles due to the prevalence of intra-molecular hydrogen bond, repulsive interactions between API molecules and strong solvation effects [256].

### 6.2. Application of Molecular Dynamics to Oral Drug Design

Oral drugs account for 60% of total drugs on the global market [257]. Oral delivery has become the most commonly used mode of drug delivery because of the advantages of continuous and controlled administration, convenient delivery and better patient compliance. The absorption of oral drugs is closely related to the drug formulation, drug physicochemical properties and the intestinal environment. Drug formulation includes dose administered, dosage form, and release rate. Drug physicochemical properties include dissociation constant (pKa), solubility, oil-water partition coefficient (logP), diffusivity, crystal morphology, intestinal enzyme metabolism, and drug-drug interactions. The intestinal environment includes physiological conditions as well as anatomical structures [258]. MD simulations can be used to predict the main parameters for optimal drug design. Dynamic Physiologically Based Pharmacokinetics (PBPK) Models are used to predict plasma drug concentration profiles for oral drug absorption. PBPK models can be classified into two types depending on the method of spatial calculation: Compartmental Models and Continuous Models.

By using Compartmental Models, Arafat et al. [259] used a combination of simulations and experiments to conduct their research. They developed a bioavailable dosage form of the poorly water-soluble drug LAB687 based on relative oral bioavailability data in dog models. Then, Arafat used GastroPlus™ software to evaluate the drug release time of formulations with different ratios of Poroxam-188, hydroxypropyl methylcellulose and magnesium stearate as controlled release matrix. Arav et al. [260] developed and validated a physiologically based continuum model. The model was used to study the absorption kinetics of levodopa from an oral controlled release formulation to reach the bloodstream. The mathematical model was validated by comparing its predictions to experimental results in healthy and Parkinsonian patients following administration in different controlled release formulation. The simulation results are identical with experimental results.

### 6.3. Application of Molecular Dynamics to Liquid, Suspension and Semisolid Dosage Forms

Ionic liquids (ILs) are complex organic salts that are widely used in industrial production, such as batteries [261,262]. Due to the cellular affinity of ILs, there are lots of biophysical and chemical-physical investigations aimed at exploiting ILs in bio-nanomedicine, drug delivery, pharmacology, and bio-nanotechnology. Some researchers used MD to explore the interaction between ILs and lipid membranes, aimed at determining the microscopic mechanisms behind their interaction [263,264,265]. In addition, ILs can be used as green solvents or active ingredients to enhance drug solubility, permeability and binding efficiency, and can be used as structuring agents in the development of nano/microparticles, including micelles, vesicles, gels, and emulsions. Kuddushi et al. [266] reviewed the application of interleukin-based gels in drug delivery. Ghaed-Sharaf et al. [267] used MD to simulate the synergistic aggregation of surface-active cations with ibuprofen drug anions in vesicles. Furthermore, ILs have shown significant potential in drug delivery, particularly for enhancing transdermal delivery. The ionic composition of ILs influences their ability to increase skin permeability. Jain et al. [268] reviewed the challenges and potential applications related to ILs in transdermal drug delivery.

It is necessary to understand the stability conditions, phase transitions and transport properties of suspension formulations. MD and quantum mechanics simulations allow the calculation of attractive-repulsive forces, macroion radial distribution functions, charge distributions and macroion-macroion forces between particles in suspension. Simulations clarify physical phenomena at the molecular level of suspensions and help optimize drug design to improve the thermodynamic stability of suspended particles [269]. Tian et al. [270] reviewed the use of MD simulations and quantum mechanical simulations to screen stabilizers and milling parameters for the preparation of nanosuspensions by wet media milling. Abdollahi et al. [271] used MD simulations to study nanosuspensions of four poorly water-soluble drugs, flurbiprofen, benzaprofen, miconazole and phenytoin sodium, using polyvinyl alcohol as stabilizer. Abdollahi explored the effect of polyvinyl alcohol on the diffusion of water molecules, providing insights into the stability of nanosuspensions.

Molecular or atomic dynamics is widely used to study the semisolid formulation stability, physicochemical properties, rheological properties, drug release rate, excipient properties, and the effect of formulation properties on drug delivery. Jia et al. [272] used a chemical enhancer method to create a low loading, high retention, easier to apply mometasone furoate cream. They used high-speed centrifugation, MD, and rheology to investigate the molecular mechanisms by which chemical enhancers increase release. The results indicated that chemical enhancers in creams can effectively reduce the cohesive energy density in the oil phase and enhance drug mobility and release. This simulation provides insights for semisolid development experiments. Vu et al. [273] investigated the stability of ternary systems composed of pure or mixed fatty alcohols, cetrimide and water using an atomic model. This ternary system was a semisolid oil-in-water system and was the main ingredient in medicated creams. The simulation results supported the hypothesis that the alkyl chain length mismatch results in a more stable mixed-alcohol system than a pure-alcohol system. There are few cases in which MD has been used to study semisolid formulations. Molecular or atomistic simulations have great value in the field of semisolid dosage forms.

## 7. Limitations and Solutions

Numerical simulation has been widely used in the field of pharmaceutical preparation. The simulation methods can predict the optimal preparation conditions, predict the quality shortcomings of the product in advance and adjust the design according to the quality. This is in line with the concept of QbD. In addition, we should focus on limitations and deviations existing in the numerical simulations so as to ensure the accuracy of the simulation results. Based on previous research in the field, we need to pay attention to the following aspects:

1. To start a numerical simulation, the first step is to develop an accurate and appropriately simplified geometric model. Non-physiological geometric models, i.e., objects, only require accurate geometric models based on material properties. But for physiological geometric models, it is important to obtain the geometric structure and parameters of physiological or pathological properties, such as nasal cavity and skin. Furthermore, physiological structures are often complex, need to be simplified and transferred into computable models to improve computational efficiency.

Taking the airway geometry model for aerosol drug delivery as an example, the drug deposition efficiency is deeply affected by the airway geometry [170], so it is crucial to establish an accurate airway model to predict the particle deposition. However, in simulations, complex airway geometry models often need to be simplified due to computational constraints. During the simplification process, special attention must be paid to the accuracy of the simplified model. Some researchers explore how to simplify airway geometry modeling while maintaining accuracy. Further, researchers also explore the effect of simplified airway geometry models on particle deposition simulations and find that the nasal vestibule plays a key role in the regional deposition of particles [168]. We should further investigate the effect of the nasal vestibule on particle deposition and how the nasal vestibule affects the deposition behavior of the downstream airway. In conclusion, physiological geometric models should be constructed by instrumental measurements and available data to improve the accuracy of the simplified model. Meanwhile, we still face many challenges in the modeling process such as the variability of lesion locations, the asymmetry of lung airways [153], and the diversity of physiological constructs. Due to the inter-individual heterogeneity of these physiological models, errors in parameter measurements, and limitations in the number of samples, we need multidimensional measurement to truly characterize all features of the physiological models. To address these issues, we need to perform extensive physiological data collection and integration analysis to build more reliable computational models that can simulate reliable and generalizable predictive data.

2. According to the motion of the object, physical parameters need to be obtained and transferred to the parameters in the numerical models. For example, fluid viscosity and density in fluid simulation; particle density and friction coefficient between particles in granule simulation, etc. The construction of the model depends on a large amount of accurate experimental data. In most of simulations, the parameters are derived from the literature, which may not be proper for the simulation to be conducted due to differences in experiments [87,230]. Therefore, to obtain accurate parameters, it could be better to design and conduct experiments for measurements.

In studies of microneedle simulation, it has been noted that a large number of parameters required for advanced modelling are often difficult to obtain [205]. For example, in a simple model of skin drug diffusion, only three parameters, partition coefficient, diffusion coefficient and path length, need to be considered. However, to further simulate the spatial variability of these parameters in the stratum corneum, the number of required parameters will increase dramatically and it is difficult to measure all of them in experiments. Due to the limitations of parameter acquisition, various calibration methods and indirect measurements are necessary to obtain the required parameters instead of direct experimental measurements. In the numerical simulation of granules, researchers find that the drug interaction parameters are difficult to measure directly through calibration methods. The same situation occurs in the numerical simulation of tablet coating, where the friction coefficient measured directly from experiment is inaccurate and thus does not accurately describe the actual state of motion of the particles. Therefore, researchers improve the accuracy of friction coefficient by measuring the dynamic angle of repose in order to improve the accuracy of the simulation [93]. In summary, in order to ensure the accuracy and reliability of the simulation model, the differences between the experimental conditions and the model parameters should be fully considered, and multiple methods should be used to obtain the required parameters.

3. The selection of the numerical technology and the setting of boundary conditions are the key prerequisites for the simulation study. Numerical technologies include solution methods for partial differential equations (FEM, FVM, FDM) and transient/steady state calculations. Boundary conditions include mesh setting, entrance/exit and wall setting, etc.

During the simulation process, researchers need to explore the suitable computational methods to be adopted. According to the simulation results, researchers need to optimize the boundary conditions and computational settings to ensure that the ideal converged solutions. To fulfill the whole process, researchers need to have knowledge in the following areas: i) they need to clearly distinguish the application scenarios of different mechanistic models; ii) they need to be proficient in using numerical simulation software; iii) they need to select appropriate physical fields and computational equations according to the specific study such as turbulence equations in fluid dynamics, contact models in particle simulation, and inter-phase coupling models in multiphase simulation. Furthermore, the mechanistic models used for numerical calculations often have several idealized characteristics and are not exactly equivalent to the real situation [31]. Therefore, there may be a certain degree of inaccuracy in the geometrical model segmentation, mesh division, and material parameter input. In the simulation, researchers may need several iterations to obtain suitable boundary conditions. In order to improve the accuracy and reliability of the simulation, researchers should pay more attention to minimize the algorithmic deviation.

4. Researchers should pay attention to the accuracy of simulation results. Therefore, it is necessary to carry out a validation of the model accuracy before the starting of simulation.

**Figure 17:**
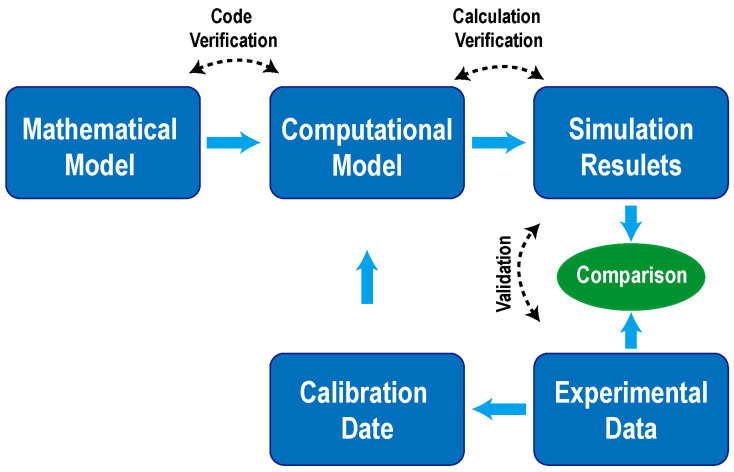
Flowchart of mechanism model application as well as validation process [70].

The validation process of the model is mainly divided into two aspects, code validation and computational validation, as shown in Figure 17. Code validation is mainly handled by the programmers of the software packages while researchers need to undertake the task of computational validation. Before starting the whole simulation process, researchers should check the reliability of the computational results by going through some of the local simulations. Ioannis et al. [70] emphasized that the core purpose of computational validation is to quantify the various potential errors, which may originate from insufficient discretization of the mesh, irrational setting of the boundary conditions, deviation of the material properties, and errors in the model generation process. Researchers usually evaluate the reliability of the simulation results by calculating a simple model before solve the full problems, or comparing the simulation results with experiment results [83,84,169]. When the validation results converge within a reasonable accuracy range, the simulation method can be further applied to solve more complex problems. However, even with validated numerical simulations, there may be some deviation among different results when the same process is computed multiple times, mainly due to the accumulation of various errors. Gregor et al. [58] also observed that although the model computations did not give exactly the same results as expected, the results showed the same qualitative trends under reasonable settings.

5. Computers have limitation on computing power. In reality, the processes of drug preparation are complex. To simulate these complex processes requires higher-capacity hardware and longer computing times. Further, it is not necessary to accurately solve the whole process of some complex problems such as drug respiratory inhalation process and granule agent particle motion process. Therefore, it is necessary to select the key steps and to consider the time cost in numerical simulations.

In applications in the field of pharmaceutical formulations, the DEM demands high computational resources and costs. The simulation time for DEMs is often two orders of magnitude larger than that of CFD [41]. In order to solve computationally intensive and process-complex problems, researchers have proposed various approaches. Firstly, researchers adopt the coupling of multiple computational models, such as the multi-scale simulation strategy proposed by Dosta et al. They used the PBM to simulate the global production process on the macroscopic scale, and CFD-DEM to calculate the kinetic properties of the particles in the granulator on the microscopic scale. Liu et al. studied nanoparticle fluidization by using CFD-DEM. Secondly, researchers can use segmented solutions for complex processes such as the simulation study of respiratory particle transport and deposition described in Section 4.3. Thirdly, researchers can use parallelization strategies to improve computational efficiency, which include CPU-GPU parallel computation [274], shared memory [275], and distributed memory based on removable hard disks. The application of these methods helps to simulate key processes of complex problems more efficiently with limited computational resources.

## 8. Outlook

The application of numerical simulation in pharmaceutics has made significant progress and is expected to play a more important role in the future. Simulation may have great prospects in the future in the field of pharmaceutical formulations, biomedicine and computing, including the integration of mechanistic and non-mechanistic simulation models, computer-assisted development of personalized formulations, macro- and microscale multiscale simulation, process simulation and optimization, and applications of interdisciplinary.

Firstly, with the development of AI, Computer Aided Drug Design (CADD), Big Data technology and Machine Learning technology, these multiple technologies are applied in a synergistic manner. The combined application of various algorithms not only helps researchers to create accurate simulation models for parameter optimization and model selection, but also significantly improves research efficiency [276,277,278]. Further, as the concept of precision medicine becomes more and more popular, the development of personalized formulations has become an inevitable trend. Under this trend, numerical simulation with its powerful capabilities has become a key force in driving the development of personalized drug treatment. By integrating multiple models such as computational fluid dynamics, pharmacokinetics and physiological pharmacokinetics (PBPK), the simulation is able to accurately calculate the complex behaviors of drugs within the individual. It provides a solid basis for personalized formulation design based on individual treatments, genetic information, physiological parameters and disease characteristics [279].

In addition, numerical simulation will develop in the direction of multi-scale in the future. From molecular simulation, mesoscopic simulation to macroscopic simulation, and from the molecular level to the cellular, tissue, organ and even holistic levels, simulation at different scales can comprehensively reveal the complex behaviors of drug during preparation, storage and use. Simulation at different scales requires the co-application of multiple simulations. At the molecular level, MD is used in the early stages of drug design. At the macroscopic level, models such as the FEM and CFD play an important role during dosage form preparation in determining the critical quality attributes and critical process parameters of the formulation. At the cellular level, the absorption and transport processes of drugs in the body can be obtained by molecular, atomic and quantum mechanical simulation. Therefore, simulation with multi-scale modelling and multi-technology combinations are valuable for application in pharmaceutics. Furthermore, numerical simulation is a new powerful interdisciplinary research tool. Further integration of numerical simulation with other disciplines such as bioinformatics, pharmaceutics, chemistry, materials science, and engineering will help to promote rapid development and breakthroughs in drug formulation.

In conclusion, the application of numerical simulation has great prospects in pharmaceutics. Simulation not only accelerates the drug development and reduces experimental costs, but also promotes the cross-application of interdisciplinary knowledge. With increasing computing power and advancement in AI and simulation technology, numerical simulation is expected to play an more critical role in design, optimization, production and quality control of drug formulations in the future.

## 9. Conclusions

Mechanistic modelling is one of the tools for the implementation of the QbD concept and plays a crucial role in drug formulation research. By effectively reducing the risk of formulation failure, computational modelling provides powerful support for the development and optimization of pharmaceutical preparations. The application of numerical simulation in the field of drug formulation not only significantly shortens the experimental cycle and reduces the economic and time costs, but also clearly reveals the physical properties and microscopic phenomena during the manufacturing process of dosage forms. The simulation results demonstrate the relationship between product design and product quality, thus realizing the core objective of QbD. Furthermore, numerical simulation is widely applicable, and can accurately simulate the physical properties of solids, liquids, gases and multiphase systems. During the simulation process, key physical properties such as velocity, pressure, temperature, concentration variations and stresses can be calculated in real time. In addition, numerical simulation can generate vector plots, contour plots, trace plots and coordinate data of the physical properties at any time, providing rich information for corresponding experiments. By processing and analyzing these data, specific functions or correlations can be established to guide the design of subsequent experiments. Application of computer simulation in pharmaceutical research will contribute to the continuous development of new drug development and pharmaceutical processes.

## Abbreviations

2D-MHF	Two-Dimensional Microhydrodynamic Focusing
3D-MHF	Three-Dimensional Microhydrodynamic Focusing
AI	Artificial Intelligence
ANN	Artificial Neural Network
API	Active Ingredient
BPM	Bonded Particle Mode
CADD	Computer Aided Drug Design
CFD	Computational Fluid Dynamics
CFPD	Computational Fluid-Particle Dynamics
CF-PD	Computational Fluid-Particle Dynamics
COF	Cross-linked Cyclodextrin Metal-Organic Framework
CT	Computed Tomography
DEM	Discrete Element Model
DPI(s)	Dry Powder Inhaler(s)
DPM	Discrete Phase Model
FDM	Finite-Difference Methods
FEM	Finite Element Method
FRR	Flow Rate Ratio
FVM	Finite Volume Method
LES	Large Eddy Simulation
MD	Molecular Dynamics
MNs:	Microneedles
MOL	Method of Line
NN	Neural Networks
PBE	Population Balance Equation
PBM	Population Balance Model
PBPK	Physiologically Based Pharmacokinetics
QbD	Quality by Design
RANS	Reynolds-averaged Navier-Stokes Equations
SIP	Stochastic Individual Path
TCPBM	Two-Compartmental Population Balance Model
TDDS	Transdermal Drug Delivery Systems
pMDI	pressurized Metered Dose Inhaler
CFC	Chlorofluorocarbon
HFA	Hydrofluoroalkane
